# The *Pleurothallis crateriformis* complex (Orchidaceae): undescribed diversity and pollination biology of a newly recognized species group from Ecuador and Peru

**DOI:** 10.3897/phytokeys.270.175070

**Published:** 2026-02-09

**Authors:** Marco M. Jiménez, Gabriel A. Iturralde, Nadia Lapo-González, Henry X. Garzón-Suárez, Gerhard Vierling, Raven Ward, Mark Wilson

**Affiliations:** 1 Grupo de Investigación en Biodiversidad, Medio Ambiente y Salud BIOMAS, Carrera de Ingeniería Agroindustrial, Facultad de Ingeniería y Ciencias Aplicadas, Universidad de Las Américas, UDLA, Vía a Nayón, Quito 170124, Ecuador Facultad de Ingeniería y Ciencias Aplicadas, Universidad de Las Américas Quito Ecuador; 2 Floplaya, Compañía de flores y plantas Yantzaza S.A., El Pangui, 190650, Ecuador Department of Organismal Biology and Ecology, Colorado College Colorado Springs United States of America; 3 Grupo Científico Calaway Dodson, Investigación y Conservación de Orquídeas del Ecuador, Quito, Ecuador Universidad Técnica Particular de Loja Loja Ecuador; 4 Herbario HUTPL, Departamento de Ciencias Biológicas, Universidad Técnica Particular de Loja, Loja, Ecuador Floplaya, Compañía de flores y plantas Yantzaza S.A. El Pangui Ecuador; 5 Bannholzweg 49/1, D – 69151, Neckargemünd, Germany Grupo Científico Calaway Dodson, Investigación y Conservación de Orquídeas del Ecuador Quito Ecuador; 6 Department of Organismal Biology and Ecology, Colorado College, Colorado Springs, CO 80903, USA Unaffiliated Neckargemünd Germany

**Keywords:** montane forests, Neotropics, northern Andes, Pleurothallidinae, taxonomy

## Abstract

A new complex of species is recognized within *Pleurothallis* subgenus *Pleurothallis* section Macrophyllae-Fasciculatae, comprising *P. crateriformis*, *P. equipedites*, *P. nipterophylla*, *P. phymatodea* and *P. pyelophera*, as well as two new species. *Pleurothallis monteroae***sp. nov**. and *Pleurothallis austrorientalis***sp. nov**. from southeastern Ecuador are described and illustrated, bringing the number of described species in the complex to seven. Localities in southeastern Ecuador are confirmed for *P. pyelophera* and *P. equipedites*, species originally described from cultivated material lacking precise collection data. Diagnostic characters distinguishing the two new species from their most morphologically similar congeners, as well as from the other five members of the complex, are presented. Summarizing the traits characteristic of the group allowed recognition of eight additional undescribed species, mostly from Peru. Unusual characteristics of the *P. crateriformis* complex include a deeply “crateriform” lip containing a nectar-like liquid, non-resupinate flowers, and extra-labellar nectar-like liquid. These attributes suggest a different pollination mechanism within *P.* sect. Macrophyllae-Fasciculatae, involving a shift from nototribic to sternotribic pollination. A possible case of morphological convergent evolution between the *P. crateriformis* complex and a group of non-resupinate *Pleurothallis* species from Mesoamerica is discussed.

## Introduction

*Pleurothallis* R.Br. sect. Macrophyllae-Fasciculatae Lindl. (1859) is the most species-rich group within the genus, comprising ~283–354 species, depending upon synonymy (Luer 2005; POWO 2025; Wilson unpubd., continuously updated list). The section is distributed from Mexico south to Bolivia and Paraguay and east to Guyana and the Antilles (Luer 2005).

In the recent past, the group was mistakenly referred to at the subsection level (e.g., Wilson et al. 2022). This taxonomic error was recognized only recently (G.A. Salazar, pers. comm.). Luer (2005) synonymized subsections Macrophyllae-Fasciculatae Luer (1988) and Cardiostolae Luer (1988) under section Macrophyllae-Fasciculatae Lindl. As a consequence, the species included in the section and the former subsection are the same; therefore, subsection Macrophyllae-Fasciculatae Luer (1988) becomes superfluous. Henceforth, we refer to the group at the higher, sectional level as *Pleurothallis* subgenus *Pleurothallis* section Macrophyllae-Fasciculatae. Phylogenomic studies are currently underway to determine whether Macrophyllae-Fasciculatae constitutes a monophyletic group (i.e., whether it includes the Cardiostolae group) and which taxonomic rank (i.e., subgenus or section) is most appropriate.

**Figure 1.**
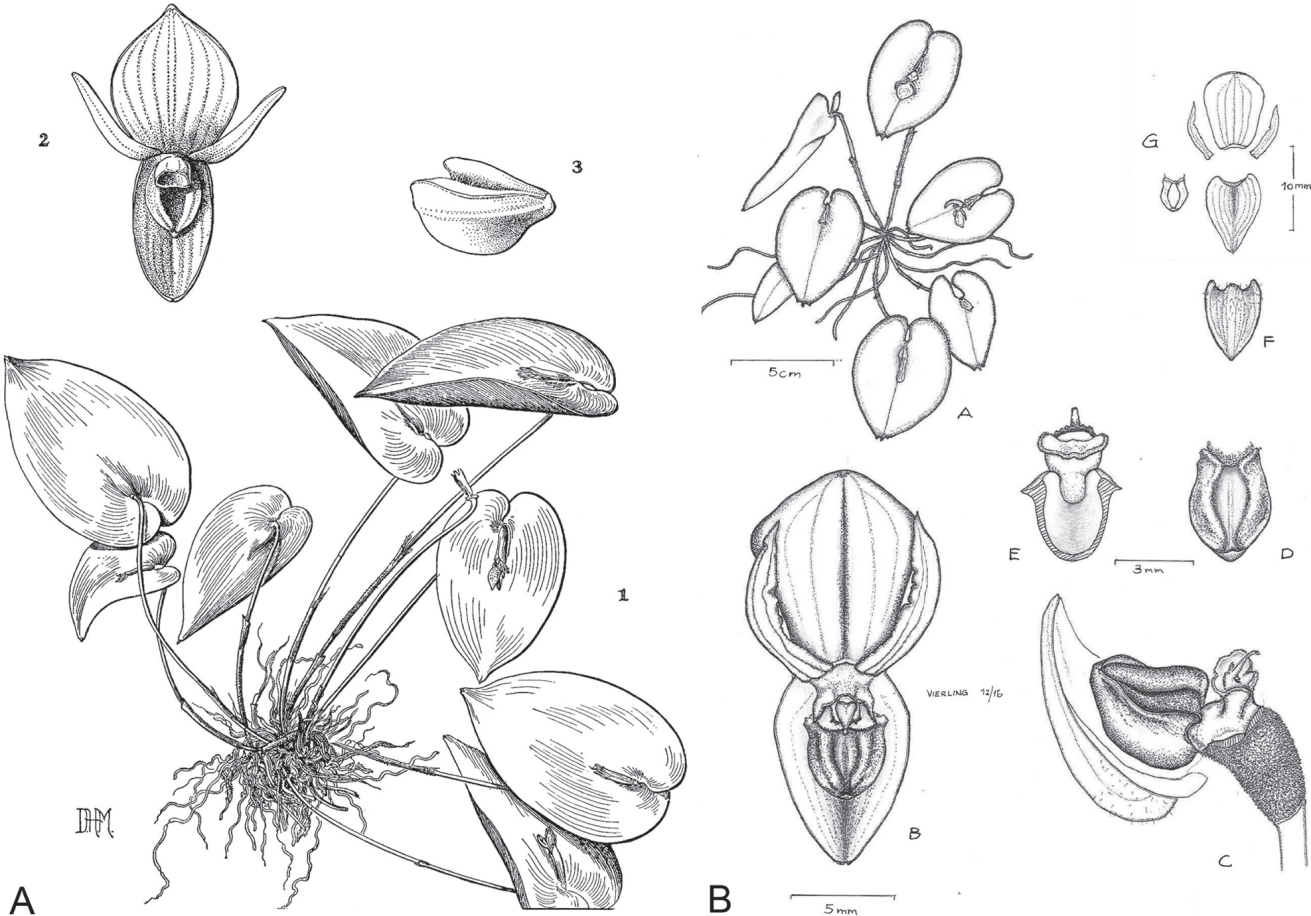
*Pleurothallis crateriformis*. **A**. Drawing from Schweinfurth (1951). **B**. Drawing by G. Vierling from a living specimen in an *ex situ* collection (this study, COCO PL1014).

**Figure 2.**
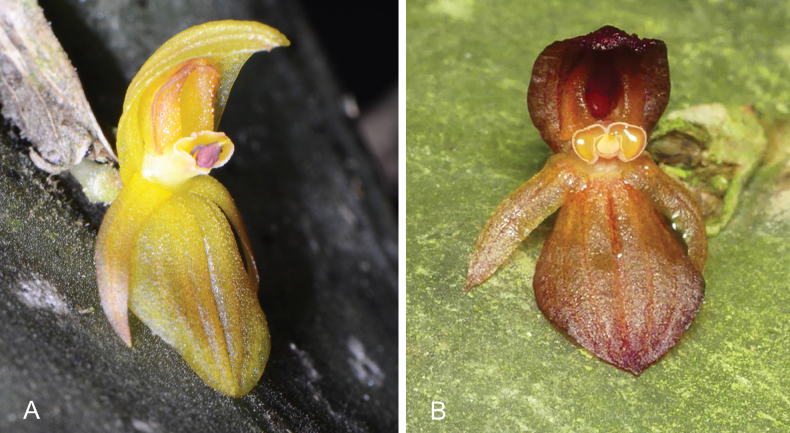
Non-resupinate flowers of **A**. *Pleurothallis crateriformis* (photo by Gerhard Vierling, *ex situ* in private collection, vouchered as COCO PL1014); **B**. *Pleurothallis pyelophera* from southeastern Ecuador (photo by Marco Jiménez).

Within this large section, several groups or complexes of morphologically similar species exist, such as the *Pleurothallis cardiostola–P. lilijae* complex (Wilson et al. 2022; Revatta-Bustos et al. 2025) and the *P. cardiothallis* complex (Pupulin et al. 2017, 2021). Here, we recognize a new complex of species sharing a suite of morphological traits with *P. crateriformis* C. Schweinf. (Figs 1, 2A) and describe two new species within that complex.

**Figure 3.**
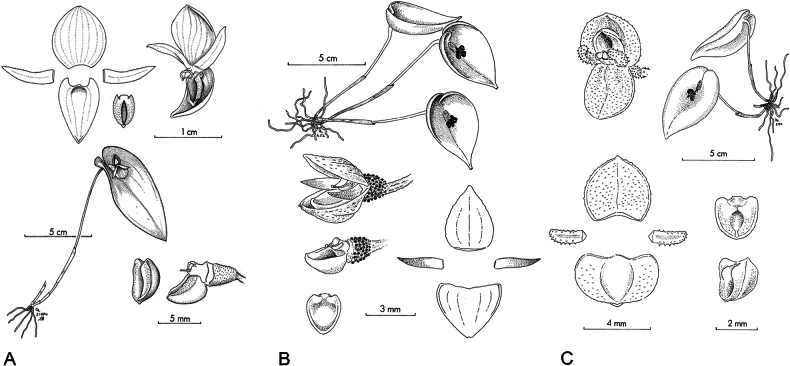
Species morphologically similar to *Pleurothallis crateriformis*. **A**. *P. pyelophera*; **B**. *P. nipterophylla*; **C**. *P. phymatodea*. Drawn by Carlyle A. Luer in: **A**. *Icones Pleurothallidinarum* XXVII (2006): pl. 256; **B**. *Icones Pleurothallidinarum* XXVII (2005): pl. 246; **C**. *Selbyana* 3(1/2) (1976): pl. 171. Courtesy of Missouri Botanical Garden Press.

*Pleurothallis crateriformis* was described from a specimen collected in the Junín Region of Peru (Schweinfurth 1951). The specific epithet refers to “*the deeply concave or bowl-shaped lip*”. *Pleurothallis pyelophera* (Luer) Pfahl is a similar species (Figs 2B, 3A), which was presumed at the time of description to be from Ecuador; its epithet is derived from the Greek “pylopher”, meaning “tub bearer”, again referring to the deeply concave lip. Both taxa share several unusual features, including highly coriaceous leaves, mostly non-resupinate flowers (see Dressler 1981 for an explanation of resupination and non-resupination), and a papillose ovary. Recognition of these shared characteristics made it apparent that several other described and undescribed species exhibit similar morphologies, including *P. nipterophylla* Luer (Fig. 3B), *P. phymatodea* Luer (Fig. 3C), and *P. equipedites* K.W. Holcomb. Together, these five species constitute what we here define as the *P. crateriformis* complex of section Macrophyllae-Fasciculatae.

**Figure 4.**
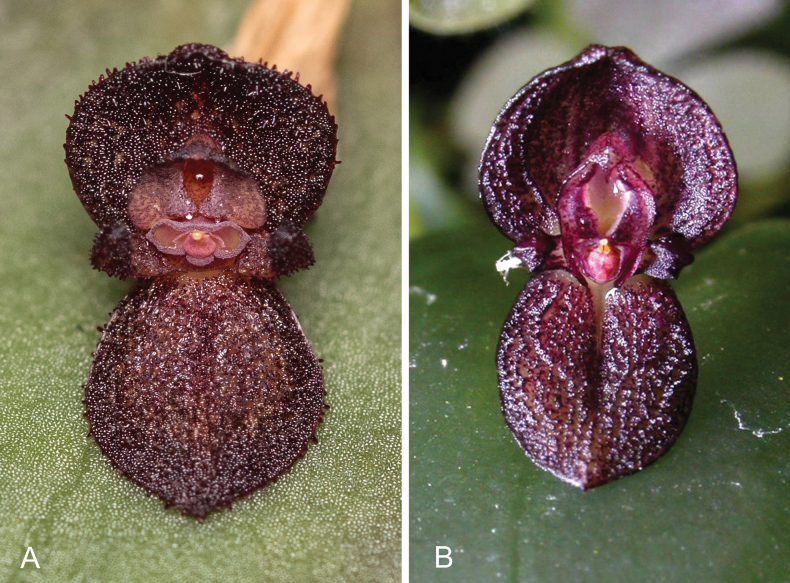
Morphological similarity between non-resupinate flowers of geographically distant species of *Pleurothallis*. **A**. *Pleurothallis phymatodea* from southeastern Ecuador (photo by Kevin Holcomb); **B**. *Pleurothallis excavata* from Central America (photo by Danny Lentz).

Interestingly, a small group of species occurs in Mesoamerica, mostly in Costa Rica and Panama, that share some of these floral morphological traits, including predominantly non-resupinate flowers and deeply “concaviform” lips (Pupulin and Zúñiga 2007). Some of these species exhibit remarkable resemblance to members of the *Pleurothallis crateriformis* complex. For example, while vegetatively quite different, the flowers of the Mesoamerican *P. excavata* Schltr. are surprisingly similar to those of *P. phymatodea* (Fig. 4). Despite this resemblance, preliminary nrITS and plastid *matK* sequencing (Wilson et al. 2013) suggests that these species are not members of section Macrophyllae-Fasciculatae, raising the possibility of convergent evolution, a common phenomenon in Pleurothallidinae (Karremans 2016).

Relatively little is known about pollination in species of section Macrophyllae-Fasciculatae. There are few recorded *in situ* observations of flower–pollinator interactions and even fewer published studies (Dodson 1962; Duque 1993; Duque-Buitrago et al. 2014; Karremans and Díaz-Morales 2019). The available information, however, suggests that species of the section are primarily pollinated by Diptera, most commonly in the families Drosophilidae, Mycetophilidae, Sciaridae, and Tephritidae (Karremans and Díaz-Morales 2019). In cases such as this, with few direct observations, one approach is to infer or hypothesize probable pollination mechanisms from floral morphology and to refine these models as additional data become available.

**Figure 5.**
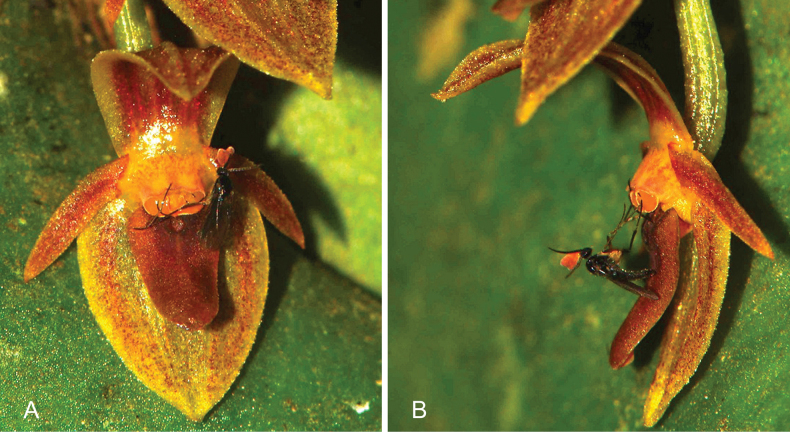
Nototribic pollinarium transfer in *Pleurothallis* sect. Macrophyllae-Fasciculatae involving resupinate flowers with a short column, apical anther, and glenion at the base of the lip. Presumed pollinator (Diptera: Sciaridae) interacting with a flower of *Pleurothallis* sp. (photos by Francisco Tobar).

The majority of species in section Macrophyllae-Fasciculatae exhibit resupinate flowers in which the lip is lowermost (Luer 2005). Frequently, the lips produce a nectar-like liquid, presumed to be a pollinator attractant or reward (for example, Wilson et al. 2022). Almost all species possess a short column that is more or less parallel to the lip, with an apical anther and flanking stigmatic surfaces (Luer 2005). In all but a few species, the lip bears a “glenion”, a small secretory structure vertically below the viscidium of the pollinarium, hypothesized to aid in positioning the pollinator for dorsal pollinarium attachment on the head of the insect (Fig. 5).

In this manuscript, we describe two new species from southeastern Ecuador belonging to the *Pleurothallis crateriformis* complex, define distinguishing characteristics of the complex based on these seven currently recognized species, document the existence of several additional undescribed species in the group, mostly from northern Peru, comment on the inferred pollination mechanism in the group, and discuss a potential case of convergent evolution with the morphologically similar group of non-resupinate *Pleurothallis* species from Mesoamerica.

## Materials and methods

### Taxonomy

The original material of the new species was compared with the original descriptions of similar *Pleurothallis* species (Schweinfurth 1951; Luer 1976, 2005, 2006; Holcomb 2023). Digital images of the holotypes and additional specimens of *Pleurothallis crateriformis*, *P. nipterophylla*, *P. phymatodea*, and *P. pyelophera* housed at COCO, MO, SEL, and UC (herbarium acronyms following Thiers 2025) were examined either in person or through JSTOR Global Plants (https://plants.jstor.org/), the iDigBio Portal (https://portal.idigbio.org/portal/search), and GBIF (https://www.gbif.org/). The iNaturalist platform (https://www.inaturalist.org) was also consulted to obtain photographic records of the species mentioned above. The new species were described using botanical terminology following Beentje (2016), with inflorescence morphology following the typology proposed by Rojas-Alvarado and Karremans (2024). Measurements of vegetative and floral parts were taken from both living material and herbarium specimens. Fresh flowers were stored in 70% ethanol and 1% glycerol to ensure preservation. Digital photographs were taken with a Panasonic FZ300 camera and Raynox DCR-250 and MSN-505 (37 mm) super macro lenses, in combination with a Yongnuo Speedlite YN560 IV flash. Figures and composite plates were prepared using Adobe Photoshop v. 25.11.0.

The area of occupancy (AOO) and extent of occurrence (EOO) were calculated with GeoCAT (Bachman et al. 2011) using a 2 × 2 km grid, based on confirmed occurrence records for each species. The conservation assessment follows the IUCN (2024) categories and criteria to propose a preliminary conservation status. The distribution map was prepared using ArcGIS Desktop 10.3 (ESRI 2024). Specimens of the new species were collected under permits MAATEDB I-CM-2022-0248 and MAATE-DNB-CM-2022-0248-M-0001, issued by the Ministerio del Ambiente y Transición Ecológica de Ecuador (MAATE), and deposited in the herbarium of the Universidad Particular de Loja (HUTPL). Geographic coordinates of the specimens were omitted for conservation purposes; detailed data can be consulted in the herbarium vouchers.

### Scanning electron microscopy

Recently opened flowers of *Pleurothallis pyelophera* (COCO accession number PL1151) were harvested from plants in the Colorado College living collection and preserved in Kew Mix (Wilson et al. 2016). Flowers were prepared for and examined by scanning electron microscopy (SEM) following methods described previously (Wilson et al. 2016, 2018). Briefly, flowers were dehydrated through an alcohol series and then critical-point dried. The specimens were mounted on aluminum stubs and sputter-coated prior to examination with the scanning electron microscope.

### Taxonomic treatment

#### Pleurothallis monteroae

Taxon classificationPlantaeAsparagalesOrchidaceae

M.M.Jiménez, F.Ramón & Mark Wilson sp. nov.

F5128955-0660-501C-9B11-B30B5FAAFC7C

urn:lsid:ipni.org:names:77376435-1

Figs 6, 7, 12 C

##### Diagnosis

**Diagnosis**. Similar to *Pleurothallis phymatodea*, from which it differs in the elliptic to ovate, acuminate leaves (vs. ovate, acute), the 5-veined, obtuse dorsal sepal (vs. 3-veined, subacute), the subacute to shortly acuminate synsepal (vs. obtuse), the ligulate, 3.9–4.6 mm long, attenuate petals (vs. oblong, ca. 2.2 mm long, obtuse); and the lip with T-shaped cavity and a glenion (vs. with a narrowly elliptic cavity without a visible glenion), the revolute margins (vs. involute), the obtuse apex (vs. rounded).

**Figure 6.**
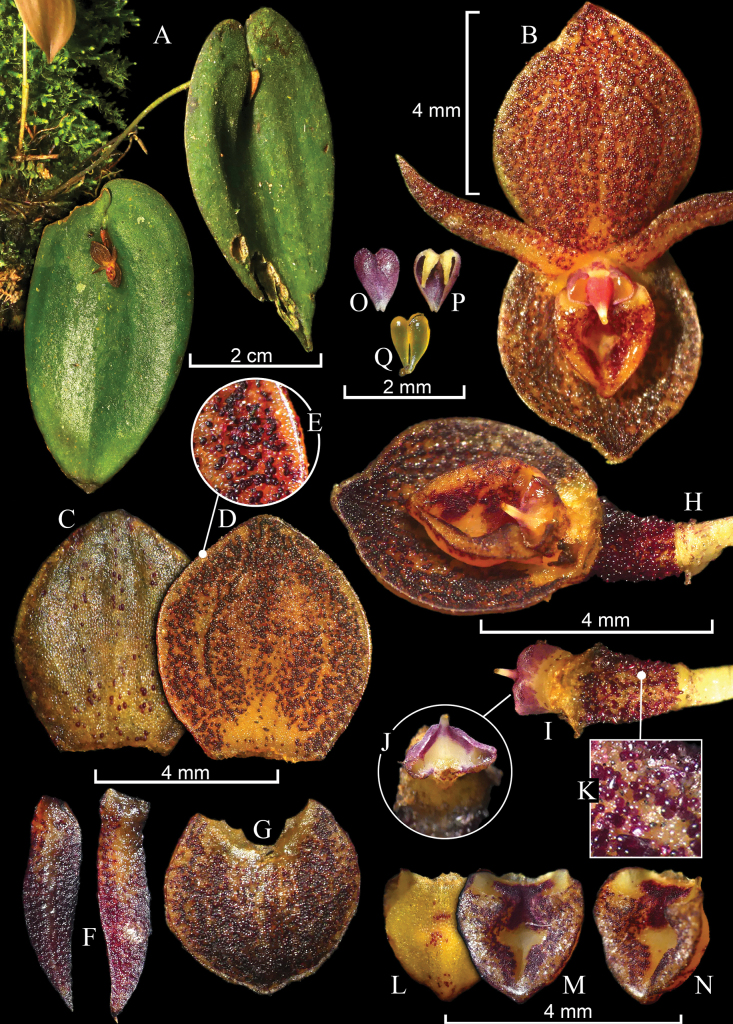
*Pleurothallis monteroae* M.M.Jiménez, F. Ramón & Mark Wilson. **A**. Habit; **B**. Flower; **C**. Dorsal sepal, abaxial view; **D**. Dorsal sepal, adaxial view with a close-up of the papillae (**E**); **F**. Petals, adaxial view; **G**. Synsepal, adaxial view; **H**. Lip, column, ovary, and synsepal, ¾ view; **I**. Column, dorsal and frontal (**J**) views with a close-up of the surface of the ovary (**K**); **L**. Lip, abaxial view; **M**. Lip, adaxial view; **N**. Lip, ¾ view; **O**. Anther cap, adaxial view; **P**. Anther cap, abaxial view; **Q**. Pollinarium. Prepared by G.A. Iturralde from photographs of the holotype.

**Figure 7.**
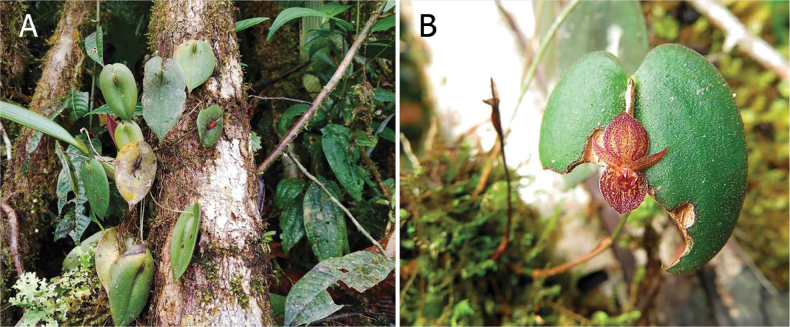
*Pleurothallis monteroae in situ*. **A**. Photo by Paola Ordóñez Montero from the type locality; **B**. Photo by Raquel Betancourt from near Tundayme, southeastern Ecuador.

##### Holotype.

Ecuador • Zamora-Chinchipe: Cerca de El Plateado, 6 August 2025, *M.M. Jiménez León & M. Jiménez Villalta* 2620 (HUTPL 15797!).

**Description**. Epiphytic herb, caespitose, to 10 cm tall. ***Roots*** slender, white, flexuous, 0.5 mm in diameter. ***Ramicauls*** suberect, green, 9.1–10.1 cm long, 0.1 cm in diameter, ascending at the base, enclosed by two papery basal sheaths at the base and one below the middle, bracts infundibuliform, upper sheath 11.7–28.7 mm long, glabrous, basal sheath 4.2–10.8 mm long, papulose. ***Leaves*** reflexed, sessile, dark green, elliptic to ovate, concave centrally between convex halves, 5.1–6.2 × 2.8–3.5 cm, microscopically papillate, subverrucose, centrally canaliculate, thickly coriaceous, with a navicular concavity behind the flower, base cordate, lobes unequal, sometimes overlapping, margins slightly revolute, apex attenuate. ***Inflorescence*** forming successive, multi-flowered coflorescences with a single open flower, subtended by a prostrate, papyraceous spathe, 4.4–5.0 mm long; pseudopeduncle short, ca. 1.6 mm long, floral bract infundibuliform, ca. 3.4 mm long. Pedicel subverrucose, 3.3–4.0 mm long, dilated at the junction with the ovary. ***Ovary*** pale yellow with purple papillae, obconic, 1.4–1.9 × 1.3–1.4 mm, coarsely papillose, furrowed. ***Flowers*** resupinate or non-resupinate, sepals, petals, and lip pale yellow or rose, profusely covered with chocolate or purple pustules, petals brown at the apex, column whitish yellow, suffused with purple above, clinandrium purple. ***Sepals*** papulose-subverrucose abaxially with sparse coarse papillae, ***dorsal sepal*** 5-veined, orbicular elliptic, 5.0–5.3 × 3.9–4.5 mm, slightly convex overall, apex obtuse; ***synsepal*** 5–7-veined, transversely elliptic, 4.3–4.6 × 3.8–4.4 mm, shallow to concave, apex subacute to shortly acuminate. ***Petals*** 1-veined, porrect, oblique, ligulate, slightly falcate, 3.9–4.6 × 0.9–1.2 mm, adaxially slightly canaliculate in the middle, margins flat, apex attenuate, slightly incurved. ***Lip*** 3-veined, cordiform-ovate, carnose, deeply concave, 2.3 × 2.0–2.1 mm, with a T-shaped cavity and a shallow, purple, inverted V-shaped, bilobed glenion at the base; base subtruncate, with short, obtuse angles; a pair of rounded calli flanking the glenion, margins slightly revolute, apex obtuse. ***Column*** complanate, 1.1 × 1.3–1.4 mm, papillose, clinandrium sinuate, rostellar flap 0.3 mm long; stigma apical, transversely elliptic, margins thickened, purple. ***Anther cap*** purple, ovate, subcordate, ca. 0.7 × 0.7 mm. ***Pollinia*** yellow, obovoid, ca. 0.7 mm long.

##### Etymology.

Named after Paola Ordóñez Montero, an enthusiastic nature photographer who observed this species.

##### Distribution and ecology.

**Figure 8.**
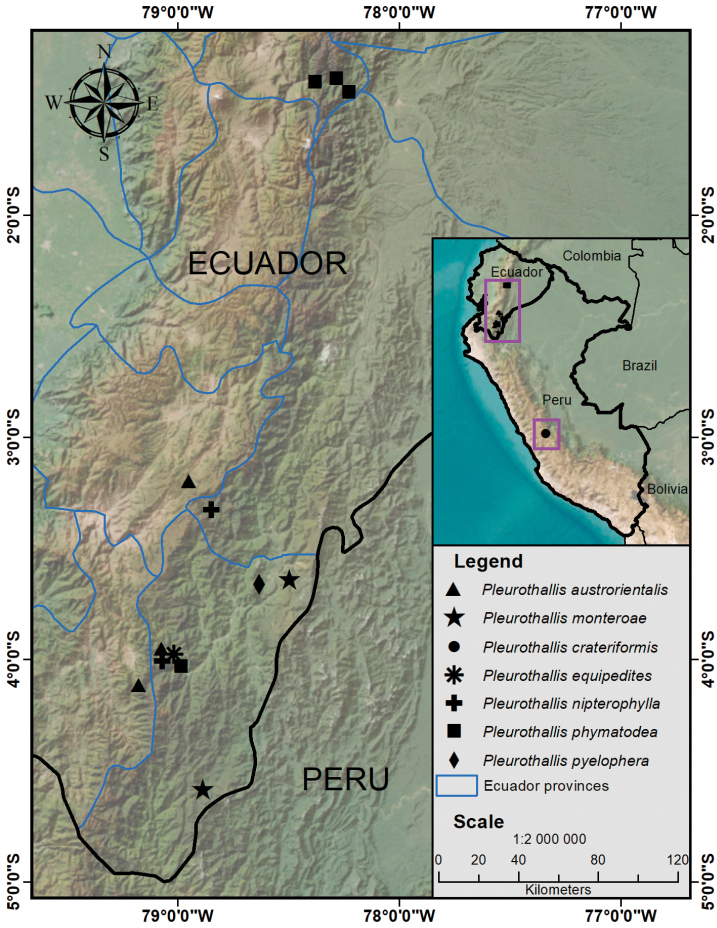
Distribution of the described species in the *Pleurothallis crateriformis* complex. Map prepared by Henry X. Garzón-Suárez.

*Pleurothallis monteroae* is known from the western foothills of the central and southern part of the Cordillera del Cóndor in the province of Zamora-Chinchipe, forming part of the ecosystem known as the evergreen lower montane forest on sandstone plateaus of the Cóndor-Kutukú ranges (BsBa03) (Ministerio de Ambiente del Ecuador 2013), at elevations between 1100 and 1700 m (Fig. 8). In the type locality, individuals of this species grow as epiphytes close to the ground in the understory. Other species of orchids, such as *Elleanthus conifer* (Rchb.f. & Warsz.) Rchb.f., *E. virgatus* (Rchb.f.) C.Schweinf., *Masdevallia lilacina* Königer, *M. mendozae* Luer, *Maxillaria pachyacron* Schltr., and *Oncidium gayi* J.M.H.Shaw, were found sympatrically with *P. monteroae*. In this area, the local flora is dominated by *Baccharis genistelloides* (Lam.) Pers., *Clusia multiflora* Kunth, *Semiramisia speciosa* (Benth.) Klotzsch, *Symbolanthus jasonii* J.E.Molina & Struwe and *Siphocampylus scandens* (Kunth) G.Don. A second locality was found north of the type locality near Tundayme by Raquel Betancourt in 2018 (Figs 7B, 8). The flowering of this species occurs between March and November.

##### Conservation assessment.

The species is currently known from four occurrences in two localities in Zamora-Chinchipe Province: three near Cerro Plateado (holotype + two iNaturalist photographic records) and one near Tundayme, ca. 100 km to the north (georeferenced photographic record). The calculated extent of occurrence (EOO) based on these records is 98 km^2^, and the area of occupancy (AOO) is 16 km^2^.

The known localities are all within an area heavily impacted by large-scale mining concessions, which have already caused severe deforestation and habitat fragmentation in the Cordillera del Cóndor region (Mestanza-Ramón et al. 2022; Mena-Quintana et al. 2025). These threats are ongoing and directly affect habitats required by orchids. Although some populations occur near government-protected areas, such as Cerro Plateado and El Quimi Biological Reserves and El Zarza Wildlife Refuge, these areas do not guarantee effective conservation. Mining concessions overlap with legally protected zones (Roy et al. 2018; Peck et al. 2024), undermining their integrity and exposing the species to severe threats. The proximity of mining concessions to all known localities of *Pleurothallis monteroae* significantly increases the risk of population decline and potential extinction. Even within protected areas, the presence of concessions indicates weak enforcement of conservation policies.

Although the species may be more widespread but under-collected in southeastern Ecuador, the extremely restricted known distribution (EOO < 5,000 km^2^; AOO < 500 km^2^; ≤ 5 locations), combined with the high level of threat and an inferred continuing decline in habitat quality, justifies a preliminary assessment of Endangered (EN) under criteria B1ab(iii)+2ab(iii), following the precautionary principle recommended by the IUCN.

##### Additional records.

**Ecuador** • **Zamora-Chinchipe**: Nangaritza, 24 April 2024, observation in iNaturalist by Paola Ordóñez Montero, https://www.inaturalist.org/observations/209190380 (Suppl. material 1); Nangaritza, 18 March 2025, observation in iNaturalist by Paola Ordóñez Montero, https://www.inaturalist.org/observations/265967102 (accessed 25 Aug 2025); near Tundayme, 2 November 2018, photo by Raquel Betancourt privately shared with M. Jiménez (reproduced in Fig. 7B).

##### Taxonomic notes.

*Pleurothallis monteroae* is most similar to *P. phymatodea* in its similarly sized plants (less than 10 cm tall), thickly coriaceous, convex leaves, a suborbicular dorsal sepal, and an ovate, bowl-shaped lip. The new species is distinguished from *P. phymatodea* by its pale yellow ovary and flowers covered with chocolate to purple papillae (vs. purple-black flowers and ovary with verrucae of the same color), petals with flat margins (vs. involute), and a transversely elliptic synsepal (vs. transversely ovate). Another difference between the two species is the transversely elliptic stigma in the new species (vs. broadly hexagonal in *P. phymatodea*; Fig. 4A). *P. monteroae* is the only species in the group known to have a glenion inside the cavity of the lip.

*Pleurothallis monteroae* also shows affinities with *P. equipedites* in its yellowish flowers with purple pustules. However, the new species differs in its smaller dorsal sepal, 5.0–5.3 × 3.9–4.5 mm, and synsepal, 4.3–4.6 × 3.8–4.5 mm (vs. 6.0–7.2 × 5.1–6.0 mm and ca. 6.7 × 6.6 mm, respectively). The most distinctive difference lies in the lip, which in *P. monteroae* is cordiform-ovate and crateriform, with a T-shaped cavity and a unique bilobed glenion surrounded by two rounded calli (vs. calceiform, with a lanceolate, canaliculate cavity and two elongated, ribbed calli).

#### Pleurothallis austrorientalis

Taxon classificationPlantaeAsparagalesOrchidaceae

M.M.Jiménez & Mark Wilson sp. nov.

E9C6D8CC-EF0A-5020-B97E-99B1D25EF92C

urn:lsid:ipni.org:names:77376558-1

Figs 9, 10

##### Diagnosis.

Similar to *Pleurothallis crateriformis*, from which it differs in the glabrous ramicaul sheaths (vs. hispid), the ovate-lanceolate leaves (vs. broadly ovate), the subterete, subverrucose ovary (vs. obconic, coarsely papillose), the elliptic-ovate, obtuse dorsal sepal (vs. round-ovate, rounded), the obovate, subacute, 7.2–7.9 mm wide synsepal (vs. oblong-ovate, obtuse, ca. 4.2 mm wide), the recurved, 7.4–8.4 × 1.3 mm, partially spreading petals (vs. incurved, 4.0 × 3.0 mm, porrect), the saccate lip with obtuse angles at the base and a rounded apex (vs. saccate-navicular with subacute angles at the base and a subacute apex), and the reniform stigma (vs. trigonous).

##### Holotype.

Ecuador • Zamora-Chinchipe: Vía Loja-Zamora, cerca de El Tambo, 2441 m, 14 April 2022, flowered in cultivation 15 February 2025, *M.M. Jiménez León & M. Jiménez Villalta* 1658 (HUTPL 15448!).

##### Description.

Epiphytic herb, caespitose, up to 19 cm tall. ***Roots*** slender, white, flexuous, 0.8 mm in diameter. ***Ramicauls*** suberect, green, 9.9–20.0 cm long, 0.2 cm in diameter, ascending at the base, enclosed by two papery basal sheaths at the base and one below the middle, bracts infundibuliform, glabrous, upper bracts 3.4–3.6 cm long, 0.2 cm in diameter. ***Leaves*** reflexed, sessile, pale green, ovate-lanceolate, concave, 7.2–10.3 × 3.2–5.4 cm, subplicate, thinly coriaceous, base cordate, lobes unequal and sometimes overlapping, margins slightly involute; apex attenuate. ***Inflorescence*** forming successive multi-flowered coflorescences with up to three open flowers, subtended by a prostrate, papyraceous spathe, 7.8–10.6 mm long; pseudopeduncle short, ca. 2.4 mm long, floral bract infundibuliform, ca. 5.3 mm long; pedicel very long, verrucose, dilated at the junction with the ovary, 10.9–12.3 mm long; ***ovary*** pale green suffused with brown to the apex, subterete, 4.3–4.7 × 1.7–1.9 mm, subverrucose-foveolate, furrowed, with black dots over the surface. ***Flowers*** resupinate or not resupinate, with sepals, petals, and lip pale green to yellow-green, adaxially covered with purple, coarse papillae (pustulate on sepals and petals and mamillate in the lip), petals apex chocolate-colored, lip whitish to the base, column whitish yellow, suffused with purple above, clinandrium purple. ***Sepals*** abaxially papillose-subverrucose, veins hollow adaxially and carinate abaxially, ***dorsal sepal*** 5-veined, elliptic-ovate, 9.5–10.0 × 7.4–8.3 mm, hollow below the middle, convex above the middle, apex obtuse; ***synsepal*** 6-veined, broadly obovate, 9.1–9.7 × 6.9.2–7.7 mm, deeply concave, apex subacute. ***Petals*** 1-veined, forward projecting, partially spreading, oblique, linear-falcate, 7.2–8.4 × 1.3 mm, convex adaxially, margins slightly involute, apex acute, recurved. ***Lip*** 3-veined, saccate, sometimes laterally complanate, 3.2–3.7 × 2.7–3.6 mm, 2.8 mm tall, elliptic to obovate-subhastate as seen from the front, with a lanceolate to narrowly ovate cavity in the middle, dilated at the base seen laterally; base subtruncate, hinged to the column-foot, with short, obtuse angles, margins slightly revolute, apex rounded. ***Column*** complanate, 3.4–3.5 × 2.2–2.4 mm, papillose in the upper half, clinandrium sinuate, rostellar flap 0.7 mm long; stigma apical, reniform. ***Anther cap*** whitish yellow, narrowly ovate, subcordate, ca. 1.4 × 0.8 mm. ***Pollinia*** yellow, narrowly obovoid, ca. 1.2 mm long.

##### Etymology.

The specific epithet austrorientalis, refers to the geographical distribution of this species in southeastern Ecuador.

**Distribution and ecology**. *Pleurothallis austrorientalis* is known from the eastern slope of the southern Ecuadorian Andes, forming part of the ecosystem known as evergreen montane forest at the south of the Cordillera Oriental of the Andes (BsMn02) (Ministerio de Ambiente del Ecuador 2013), between 2400 and 2700 m in elevation. This region corresponds to the southern Ecuadorian provinces of Azuay, Loja, and Zamora-Chinchipe (Fig. 8), which is known as a biological ‘hotspot’ due to its extraordinarily high diversity and endemism levels (Quizhpe et al. 2002; Tapia-Armijos et al. 2015). This area is the weather divide between the humid Amazon (“Oriente”) and the dry Inter-Andean region, and it is the transition zone between the perhumid montane broad-leaved forest and the upper montane forests (Beck et al. 2018).

Individuals of this species grow as epiphytes close to the ground in the understory over ravines. The flowering of this species occurs between February and May. It grows together with other orchid species such as *Cyrtochilum funis* (F.Lehm. & Kraenzl.) Kraenzl., *Masdevallia mandarina* (Luer & R.Escobar) Luer, *M. norops* Luer & Andreetta, *Maxillaria hastulata* Lindl., *Pleurothallis bivalvis* Lindl. and *P. omoglossa* Luer.

##### Conservation assessment.

The species is known from four occurrences representing four localities: Zamora-Chinchipe Province (holotype near El Tambo), one record near Estación Científica San Francisco, approximately 3 km to the south, one near Cajanuma (approximately 20 km to the south), and Azuay Province (near Jima, approximately 85 km to the north). The calculated extent of occurrence (EOO) of *Pleurothallis austrorientalis* is 430 km^2^, and the area of occupancy (AOO) is 16 km^2^.

Studies have documented a pronounced trend of habitat loss and fragmentation in the southeastern provinces of Ecuador. Tapia-Armijos et al. (2015) reported that between 1976 and 2008, the region experienced annual deforestation rates ranging from 0.75% to 2.86%, accompanied by a drastic reduction in the average size of forest fragments and increased isolation. Approximately 46% of the original forest has been converted into landscapes dominated by agricultural activities, compromising ecological connectivity and ecosystem resilience. This fragmentation is not restricted to unprotected areas; even within the National System of Protected Areas (SNAP) and protective forests, patterns of forest cover loss and declining structural and functional connectivity have been observed (Kleemann et al. 2022; Noh et al. 2022). The expansion of pastures and crops remains the main driver of fragmentation in Andean and Amazonian ecosystems, compounded by external pressures such as road construction and mining (Mestanza-Ramón et al. 2022; Mena-Quintana et al. 2025).

**Figure 9.**
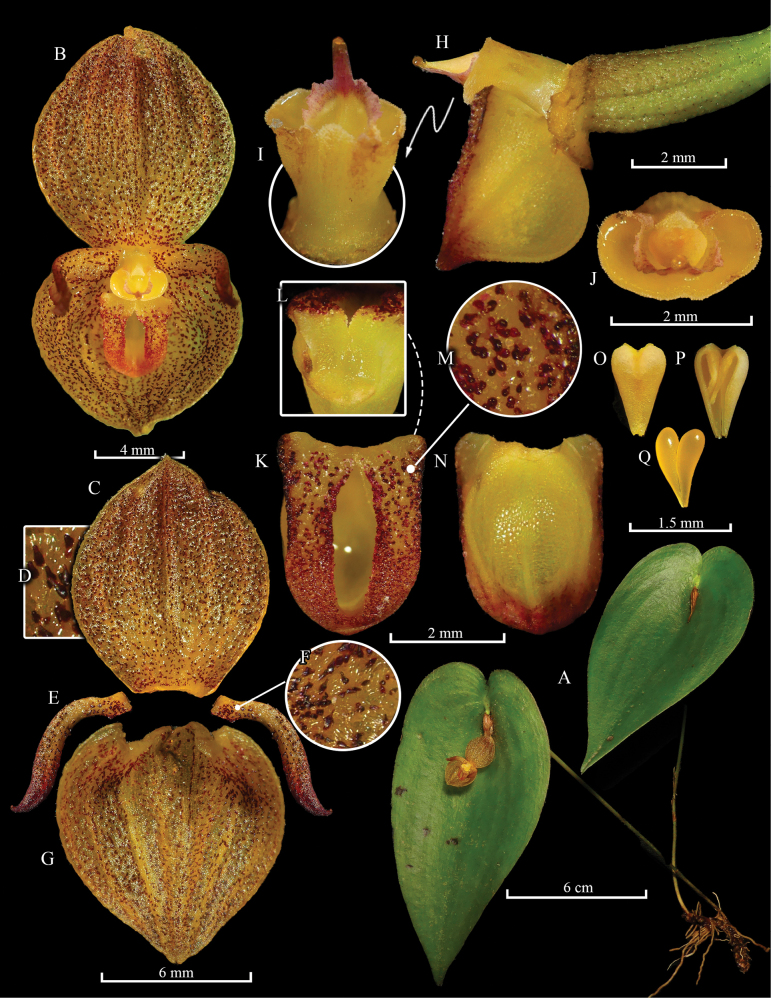
*Pleurothallis austrorientalis* M.M.Jiménez & Mark Wilson. **A**. Habit; **B**. Flower; **C**. Dorsal sepal, adaxial view with a close-up of the pustules (**D**); **E**. Petals, adaxial view with a close-up of the pustules (**F**); **G**. Synsepal, adaxial view; **H**. Lip, column, and ovary in lateral view with a close-up of the column in dorsal view (**I**); **J**. Frontal view of the column; **K**. Lip in adaxial and dorsal (**L**) views with a close-up of the surface (**M**); **N**. Lip, abaxial view; **O**. Anther cap, adaxial view; **P**. Anther cap, abaxial view; **Q**. Pollinarium. Prepared by N. Lapo-González from photographs of the holotype.

Given the restricted known distribution (EOO < 5,000 km^2^; AOO < 500 km^2^; ≤ 5 locations) and the documented habitat degradation, we recommend assessing *Pleurothallis austrorientalis* as Endangered (EN) under criteria B1ab(iii)+2ab(iii).

##### Additional specimens examined.

**Ecuador** • **Azuay**: Near Jima, cultivated by Linder Suin, 6 Mar 2001, *C. Luer* 19773 (SEL! Flower in alcohol); “Zamora-Chinchipe” [Loja]: Cajanuma Range south of Loja, 2750 m, 21 Mar 1985, *C. Luer, J. Luer, A. Hirtz & W. Flores* 10739 (SEL 47763! Flower in alcohol; MO 1241799!); Zamora-Chinchipe: Area of Estación Científica San Francisco, road Loja-Zamora, ca. 35 km from Loja, 2400 m, 12 May 2006, *F. Werner & A. Zach* 1991 (SEL 54501!); purchased in Europe from unknown vendor as *Pleurothallis crateriformis*, of unknown origin, flowered in cultivation in Zamość, Poland, 19 Mar 2024, *M. Gorbuz, G. Gorbuz & M. Wilson s.n*. (flowers in spirits, COCO PL1257!).

##### Taxonomic notes.

**Figure 10.**
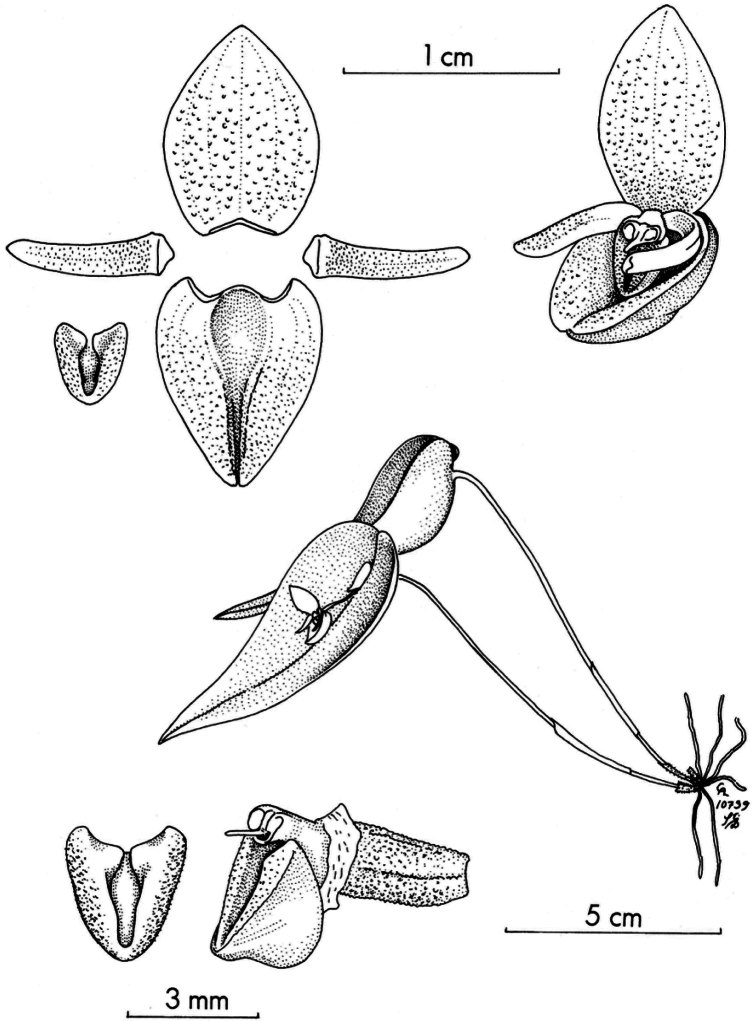
*Pleurothallis austrorientalis* illustrated by Carlyle A. Luer as *Acronia crateriformis* in *Icones Pleurothallidinarum* XXVII (2005): pl. 227. Courtesy of Missouri Botanical Garden Press.

*Pleurothallis austrorientalis* shares with *P. crateriformis* and *P. pyelophera* a deeply concave lip that lacks calli and is ventrally ventricose. The former occurs in central Peru. The differences between *P. austrorientalis* and *P. crateriformis* were outlined in the diagnosis; however, additional distinctions are noted here. The new species has previously been confused with *P. crateriformis*. Luer (2005), in his monograph on *Acronia* C. Presl section Macrophyllae-Fasciculatae, illustrated the species and misidentified it as *Acronia crateriformis* (Fig. 10). *Pleurothallis austrorientalis* is distinguished from *P. crateriformis* by its concave-subplicate leaves attenuate at the apex (vs. plane, acute), the adaxial surface of the flowers covered with chocolate-colored papillae (vs. yellow), linear-falcate petals with revolute margins (vs. lanceolate-linear, flat), a broadly obovate, crateriform, subacute synsepal, 7.2–7.9 mm wide (vs. navicular, oblong-ovate, obtuse, 4.2 mm wide), a saccate lip (vs. navicular-saccate), and a whitish yellow anther (vs. purple).

From *Pleurothallis pyelophera*, the new species is distinguished by its larger, attenuate leaves, 7.2–10.3 cm long (vs. 5.5–7.0 cm long, acute), a subverrucose, subterete ovary (vs. densely, minutely papillose, obconic), uniformly colored flowers (vs. striped), an elliptic-ovate, 5-veined dorsal sepal (vs. 7–8-veined, subcircular), a bowl-shaped synsepal (vs. navicular), linear-falcate, 1-veined petals with revolute margins (vs. 2-veined, oblong, margins flat), a lip that is elliptic-saccate with a narrowly elliptic cavity (vs. thickly navicular with a narrowly ovate cavity), obtuse basal angles (vs. triangular), a rounded apex (vs. obtuse), and the reniform stigmatic cavity (vs. trapeziform).

### Additional species of *Pleurothallis* examined

#### Pleurothallis crateriformis

Taxon classificationPlantaeAsparagalesOrchidaceae

C.Schweinf. (1951: 88, t. 26).

75581CF5-3FFC-5A6D-82B0-42E7A69DD071

Acronia crateriformis (C. Schweinf.) Luer (2005: 120). Synonyms.
Zosterophyllanthos crateriformis (C. Schweinf.) Szlach. & Kułak (2006: 188).

##### Specimen examined.

**Peru • Junín**: Tarma, Agua Dulce, on a tree in low highland forest, 2000 m, 17 March 1948, *F. Woytkowski 35487* (Holotype: UC-1217130!); Purchased in Europe from an unknown vendor, of unknown origin, flowered in cultivation in Neckargemünd, Germany, 13 Dec 2017, *G. Vierling & M. Wilson s.n*. (flowers in spirits, COCO PL1014!).

#### Pleurothallis equipedites

Taxon classificationPlantaeAsparagalesOrchidaceae

K.W.Holcomb (2023: 1).

F541E8B4-9C11-5516-A47B-422ADB129D38

##### Holotype.

Ecuador • Without collection data. K.W. Holcomb 18306 (Holotype: GEO).

##### Additional specimen examined.

**Figure 11.**
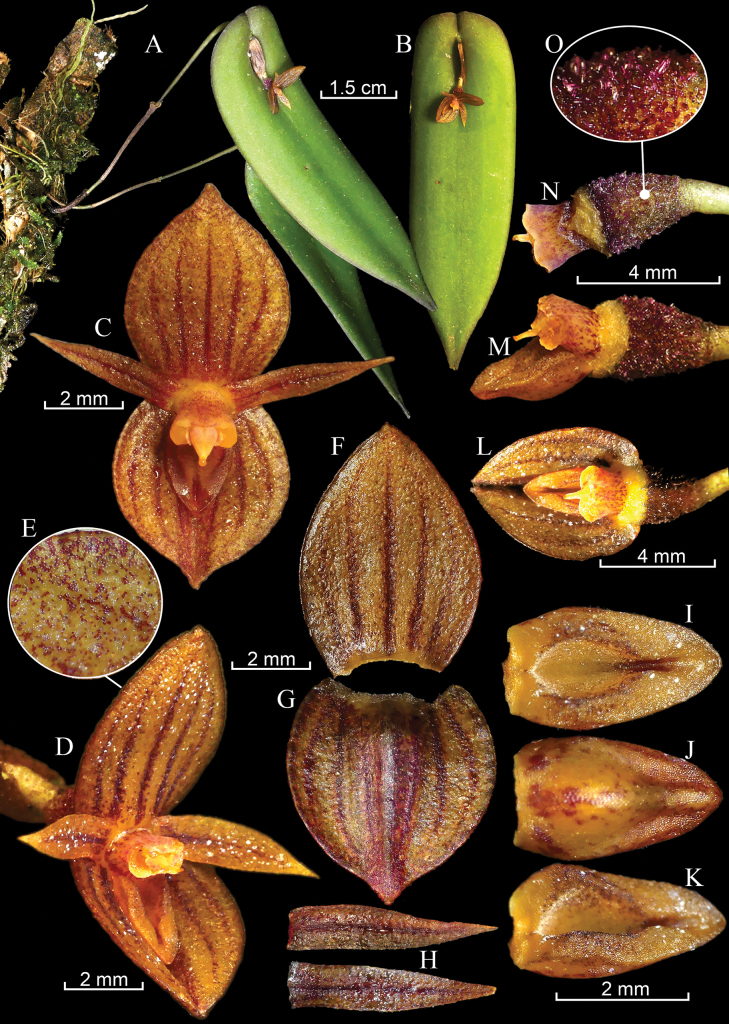
*Pleurothallis equipedites* K.W. Holcomb. **A**. Habit; **B**. Leaf; **C**. Flower, frontal view; **D**. Flower, ¾ view with a close-up of the adaxial surface of the sepals (**E**); **F**. Dorsal sepal; **G**. Synsepal, adaxial view; **H**. Petals; **I**. Lip, dorsal view; **J**. Lip, ventral view; **K**. Lip, ¾ view; **L**. Lip, column, ovary, and synsepal, dorsal view; **M**. Lateral view of the column, lip, and ovary; **N**. Dorsal view of the column, lip, and ovary, with a close-up of the surface of the ovary (**O**). Prepared by G.A. Iturralde based on M.M. Jiménez & M. Jiménez Villalta *2650 and 2651*.

**Figure 12.**
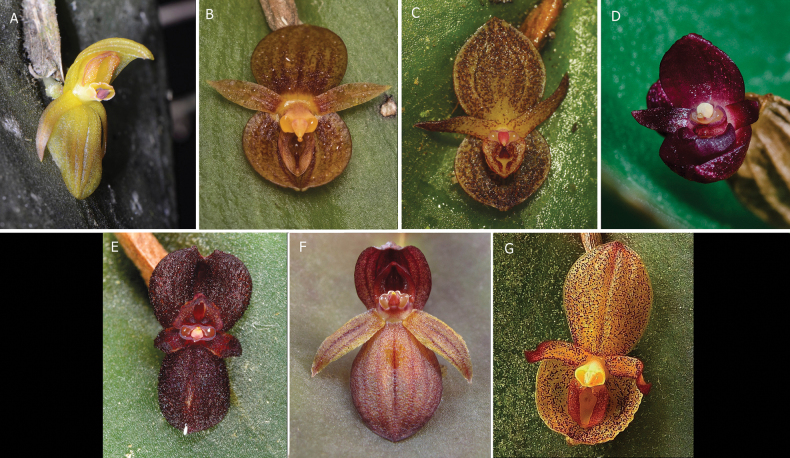
Species in the *Pleurothallis crateriformis* complex. **A**. *P. crateriformis* (*ex situ* collection, photo by Gerhard Vierling); **B**. *P. equipedites* (*ex situ* collection, photo by Kevin Holcomb); **C**. *P. monteroae* (southeastern Ecuador, photo by Marco Jiménez); **D**. *P. nipterophylla* (*ex situ* collection, photo by Pontus Aratoun); **E**. *P. phymatodea* (*ex situ* collection, photo by Ron Parsons); **F**. *P. pyelophera* (*ex situ* collection of Ecuagenera, photo by Mark Wilson); **G**. *P. austrorientalis* (*ex situ* collection, photo by Grzegorz Gorbuz). Figure prepared by Marco M. Jiménez.

**Ecuador • Zamora-Chinchipe**: vía Loja-Zamora, cerca de El Tambo, 1604 m, 7 September 2025, *Marco M. Jiménez & M. Jiménez Villalta 2651* (HUTPL 15799!) (Figs 11, 12B).

##### Notes.

*Pleurothallis equipedites* was originally described in 2023 from a cultivated plant of unknown origin by Kevin Holcomb (Holcomb 2023), together with a photograph by R. Parsons of a plant found along a roadside between Loja and Zamora.

Here, we confirm the presence of this species in Zamora-Chinchipe Province, southeastern Ecuador. Flowering individuals were found, photographed, and collected at the confirmed locality. Both dark- and pale-flowered forms were observed in this species.

#### Pleurothallis nipterophylla

Taxon classificationPlantaeAsparagalesOrchidaceae

Luer (1976: 148).

5D3C0C73-0A9D-5C2C-84A0-D637F5727BF5

Synonyms. Acronia nipterophylla (Luer) Luer (2005: 163).
Zosterophyllanthos nipterophyllus (Luer) Szlach. & Kułak (2006: 190).

##### Specimens examined.

**Ecuador • Zamora-Chinchipe**: Epiphytic in forest along the new road between Loja and Zamora, 2250 m, 22 May 1988, *Luer et al. 13586* (MO-1244995!); Epiphytic in scrubby vegetation beside the road near km 35 between Loja and Zamora, ca. 1500 m, collected 5 August 1975, flowered in cultivation 14 February 1976 m, *C. Luer, G. Luer & S. Wilhelm 656* (Holotype: SEL-000862!); between Loja and Zamora, near km 20, collected 11 February 1978, flowered in cultivation 1 May 1978, *C. Luer, J. Luer & M. Portilla 2906* (SEL-55068!); Area of Estación Científica San Francisco, road Loja-Zamora, ca. 35 km from Loja, at entrance to David Neill’s 1 ha plot (off transect 2), 2100 m, 12 May 2005, *F. Werner & D. Armijos 2593* (SEL-55067!).

##### Additional record.

**Ecuador • Morona-Santiago**: Gualaquiza, 2240 m, 20 February 2025, observation in iNaturalist by Luis Salagaje, https://www.inaturalist.org/observations/262465969 (Suppl. material 2).

#### Pleurothallis phymatodea

Taxon classificationPlantaeAsparagalesOrchidaceae

Luer (1976: 170).

664A8E1B-144A-558F-A5AD-066463BF4FDA

Synonyms. Acronia phymatodea (Luer) Luer (2005: 176).
Zosterophyllanthos phymatodeus (Luer) Szlach. & Kułak (2006: 192).

##### Specimens examined.

**Ecuador • Tungurahua**: Epiphytic in guava trees near Rio Negro, 1500 m, 28 Mar 1984, *C. Luer et al. 9776* (MO-1244974!); between Río Blanco and Río Verde along cliff face, on road from Baños to Puyo, 1800 m, 7 Jan 1962, *C. Dodson & L. Thien 1961* (SEL-01907!); about 10 km east of Baños along the Pastaza River, 1800 m, 18 Mar 1976, *C. Luer, J. Luer & P. Taylor 890* (Holotype: SEL-000877!); Zamora-Chinchipe: Loja-Zamora, km 40, 1500 m, 31 Aug 1975, *C. Luer, G. Luer & S. Wilhelm 609* (SEL-055207!).

##### Additional record.

**Ecuador • Tungurahua**: Baños de Agua Santa, 1540 m, January 2022, observation in iNaturalist by Nolan Exe, https://www.inaturalist.org/observations/108211686 (Suppl. material 3).

#### Pleurothallis pyelophera

Taxon classificationPlantaeAsparagalesOrchidaceae

(Luer) Pfahl (2013: 1).

346D230C-00F1-5D0C-8FCE-8D58864BFB7C

Acronia pyelophera Luer (2016: 247, f. 3.). Basionym.

##### Holotype.

Ecuador • Morona-Santiago: Without collection data, collected by Ecuagenera, cultivated in Howell, MI, Feb. 2006, L. O’Shaughnessy 3233 (Holotype: MO-2490851!).

##### Additional specimen examined.

**Ecuador • Zamora-Chinchipe**: Cerca de Pachicutza, 1450 m, 26 September 2025, *M. Jiménez 2669* (HUTPL!).

##### Notes.

*Pleurothallis pyelophera* was originally described as *Acronia pyelophera* Luer by Luer (2006) from an Ecuadorian specimen without a known locality, collected by Ecuagenera, presumably in Morona-Santiago Province, and subsequently imported and cultivated in Howell, Michigan, United States.

Here, we confirm the presence of this species in Zamora-Chinchipe Province, southeastern Ecuador. Flowering individuals were found, photographed, and collected at the confirmed locality. Both dark- and pale-flowered forms were observed in this species.

## Discussion

### Taxonomy

Within *Pleurothallis* subgenus *Pleurothallis* section Macrophyllae-Fasciculatae, taxonomists have recognized two groups of morphologically similar species: the *P. cardiothallis* complex (Pupulin et al. 2017, 2021) and the *P. cardiostola–P. lilijae* complex (Wilson et al. 2022; Revatta-Bustos et al. 2025). Herein, we propose a third group, the *P. crateriformis* complex. Each of these three complexes is composed of species that share a subset of morphological traits used to characterize the group. As a result, the composition of each complex is subjective and may vary according to taxonomic interpretation. The complexes may represent phylogenetic lineages; however, as with other infrageneric groupings within *Pleurothallis*, confirmation of evolutionary relatedness awaits completion of ongoing phylogenomic studies (Arias, Eserman & Wilson, unpubd.). The set of traits characterizing the *P. crateriformis* complex includes small- to medium-sized caespitose herbs (less than 20 cm tall); cordate, mostly ovate, reflexed, highly coriaceous leaves; reclined spathes; non-resupinate flowers; flowers and ovaries that are coarsely papillose, papulose, or verrucose; narrow, mostly falcate petals; a cucullate synsepal; and a deeply concave, saccate, or “crateriform” lip. The seven described species in the complex—*P. crateriformis*, *P. equipedites*, *P. monteroae*, *P. nipterophylla*, *P. phymatodea*, *P. pyelophera*, and *P. austrorientalis* (Fig. 12)—appear to be geographically distributed from central-eastern Ecuador to central Peru (Fig. 8). These species are readily distinguished based on floral morphology and dimensions (Table 1).

**Table 1.** Summary of the principal differences between species of the *Pleurothallis crateriformis* complex.

Species	*Pleurothallis monteroae*	*Pleurothallis austrorientalis*	*Pleurothallis crateriformis*	*Pleurothallis equipedites*	*Pleurothallis nipterophylla*	*Pleurothallis phymatodea*	*Pleurothallis pyelophera*
Plant (height)	10 cm	19 cm	16 cm	10 cm	13 cm	9 cm	14 cm
Leaves	Elliptic- ovate, convex, 5.1–6.2 × 2.8–3.5 cm, apex attenuate	Ovate-lanceolate, concave, 7.2–10.3 × 3.2–5.4 cm, apex attenuate	Broadly ovate, flat, 6.6–8.0 × 4.8–5.0 cm, apex acute	Narrowly ovate to oblong, convex, 6.2–11.7 × 2.25–3.0 cm, apex attenuate	Ovate, deeply concave, 5.0–5.5 × 3.2–3.8 cm, apex acute	Ovate, convex, 3.0–7.0 × 2.0–4.0 cm, apex acute	Ovate, concave, 5.5–7.0 × 3.5–4.0 cm, apex acute
Ovary	Coarsely papillose, 1.4–1.9 × 1.3–1.4 mm, obconic	Subverrucose with black dots, 4.3–4.7 × 1.7–1.9 mm, subterete	Coarsely papillose, ca. 4.5 × 4.0 mm, obconic	Coarsely papillose, ca. 2.2 × 2.2 mm, obconic	Coarsely papillose, ca. 1.5 × 1.5 mm, obconic	Verrucose, 2.0–3.0 mm long, obconic	Densely minutely papillose, 3.5 × 2.8 mm, obconic
Flowers	Resupinate, pale yellow with purple pustules, ca. 0.9 × 0.8 cm	Resupinate, pale green to yellow-green with purple coarse papillae, ca. 2.0 × 1.0 cm	Non-resupinate, yellow-green with yellow papillae, ca. 1.9 × 0.9 cm	Resupinate, amber colored with purple stripes and pustules, ca. 1.4 × 1.2 cm	Resupinate, dark purple, paler to the base of sepals and petals, ca. 0.4 × 0.3 cm	Non-resupinate, purple-black, including the verrucae, 0.7 × 0.4 cm	Non-resupinate, pale green and brown with dark brown veins and papillae, 1.8 × 1.2 cm
Dorsal sepal	5-veined, orbicular-elliptic, 5.0–5.3 × 3.9–4.5 mm, apex obtuse	5-veined, elliptic-ovate, 9.2–10.0 × 7.2–7.9 mm, apex obtuse	5-veined, round-ovate, ca. 8.0–6.9 × 7.1 mm	5-veined, broadly ovate to orbicular, 6.0–7.2 × 5.1–6.0, apex subacute	3-veined, ovate, concave, 4.0 × 3.0 mm, apex obtuse to subacute	3-veined, broadly ovate to orbicular, 4.0–5.0 × 4.0–5.0 mm, apex subacute	7–8-veined, subcircular, ca. 10.0 × 9.0 mm, apex obtuse
Synsepal	5–7-veined, transversely elliptic, 4.3–4.6 × 3.8–4.5 mm, shallow, apex subacute	6-veined, broadly obovate, 9.2–10.0 × 7.2–7.9 mm, crateriform, apex subacute	6-veined, oblong-ovate, ca. 8.5–4.2 mm, navicular, apex obtuse	7-veined, ca. 6.7 × 6.6 mm, broadly elliptic, scutellate, apex subacute, bifid	4-veined, 3.5 × 4.0 mm, ovate, deeply concave, apex obtuse	6-veined, transversely ovate, 3.0–4.0 × 5.0–6.0 mm, concave, apex obtuse	6-veined, ovate, ca. 11.0 × 6.5 mm, navicular, apex subacute
Petals	1-veined, porrect, ligulate, slightly falcate, 3.9–4.6 × 0.9–1.2 mm, margins flat, apex attenuate	1-veined, partially spreading, linear, falcate, 7.4–8.4 × 1.3 mm, margins revolute, apex acute-recurved	1-veined, porrect, lanceolate-linear, falcate, ca. 4.0 × 3.0 mm, margins flat, apex subacute	1-veined, spreading, lanceolate, slightly falcate, 4.0–5.2 × 1.0–1.4 mm, margins flat, apex attenuate	1-veined, partially spreading, oblong, falcate, ca. 3.0 × 0.8 mm, margins involute, apex acute	1-veined, partially spreading, oblong, falcate, ca. 2.2 × 0.8 mm, margins involute, apex obtuse	2-veined, porrect, oblong, falcate, ca. 9.0 × 2.0 mm, margins flat, apex acute
Lip	Cordiform-ovate, crateriform, 2.3 × 2.0–2.1 mm, with a T-shaped cavity, basal angles obtuse with two rounded calli surrounding the glenion, apex obtuse	Elliptic-saccate, ventricose behind, 3.2–3.7 × 2.6–2.9 mm, with a narrowly elliptic to ovate cavity, basal angles obtuse, without calli near the cavity, apex rounded	Ovate-navicular, ventricose behind, 4.0 × 3.0 mm, with a narrowly ovate cavity, basal angles subacute, without calli near the cavity, apex subacute	Calceiform, 3.0–3.1 × 1.9–2.0, with a lanceolate cavity that is centrally canaliculate, basal angles obtuse, with two elongated, ribbed calli near the cavity, apex obtuse	Ovoid, crateriform, 2.5 × 2.25 mm, with a deeply concave, ovate cavity, the base thickened with a pair of marginal calli near the cavity, apex obtuse	Ovoid, crateriform, 2.0 × 2.0 mm, with a narrowly elliptic cavity, basal angles rounded with two rounded calli near the cavity, apex rounded	Thickly navicular, ventricose behind, 5.5 × 2.7 mm, with a narrowly ovate cavity, basal angles triangular, without calli near the cavity, apex obtuse
Column	Ca. 1.1 × 1.4 mm, stigma transversely elliptic	3.4–3.5 × 2.2–2.4 mm, stigma reniform	Ca. 3.5 × 3.2 mm, stigma trigonous	Ca. 2.5 × 2.2, stigma broadly hexagonal with round sides	Ca. 1.0 × 1.0 mm, stigma transversely elliptic	Ca. 1.0 × 2.0 mm, stigma broadly hexagonal	Ca. 2.0 × 3.0, stigma trapeziform
Anther cap color	Reddish-brown	Whitish yellow	Brown	Whitish yellow	Whitish yellow	Whitish purple	Whitish yellow

**Figure 13.**
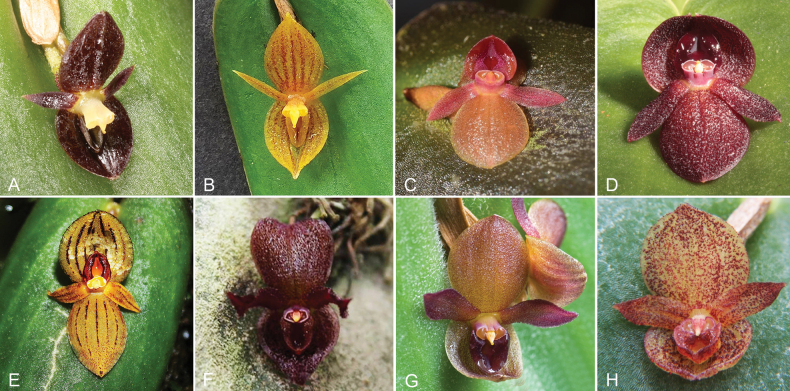
As yet undescribed species in the *P. crateriformis* complex. **A**. *P. aff. equipedites* (*ex situ* collection, photo by Duane McDowell); **B**. *P. aff. equipedites* (*ex situ* collection, photo by Maria & Grzegorz Gorbuz); **C**. *P. aff. crateriformis* (*in situ* in southeastern Ecuador, photo by Gilberto Merino); **D**. *P. aff. crateriformis* (*ex situ* collection, photo by M. Wilson); **E**. *P. aff. crateriformis* (*in situ* near Cusco, Peru, photo by Leo Huillca); **F**. *P. aff. crateriformis* (*in situ* near Cajamarca, Peru, photo by Luis Ocupa Horna); **G**. *P. aff. crateriformis* (*ex situ* collection, photo by Ecuagenera); **H**. *P. aff. monteroae* (*ex situ* collection, photo by Wiel Driessen).

As mentioned above, the species composition of the complex is somewhat subjective, based on possession of a subset of a list of morphological traits. One trait shared by all seven species is a noticeably verrucose or papillose ovary. Other species in section Macrophyllae-Fasciculatae possess somewhat verrucose or papillose ovaries, but *Pleurothallis lynniana* (Luer) Pfahl also exhibits two other traits, non-resupinate flowers and extra-labellar nectar-like liquid (see below), and may be a candidate for the complex despite the absence of a crateriform lip. Further, examination of iNaturalist observations and photographs of species in public and private collections has revealed eight additional, as yet undescribed species that could be members of the complex (Fig. 13), all, as far as can be ascertained, distributed from central-eastern Ecuador to central Peru. Future studies will endeavor to describe as many of these species as possible, confirm species distributions, further characterize the traits shared by the complex, and attempt to determine whether the complex represents a lineage of evolutionarily related species.

### Pollination biology

The *Pleurothallis crateriformis* complex species exhibit three characteristics that are of interest to the pollination biology of this group: the deeply concave lip; the secreted liquid on the sepals and petals; and the non-resupinate flowers in some of the species.

**Figure 14.**
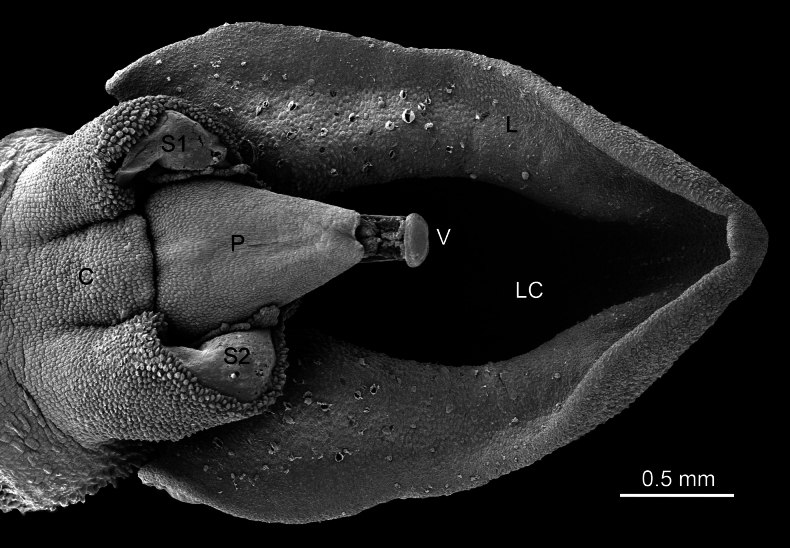
Scanning electron micrograph of the column, anther, and lip of *Pleurothallis pyelophera* COCO PL1151 by Raven Ward. (C = column; P = pollinarium covered by anther cap; S1 and S2 = stigmatic surfaces; V = viscidium; L = lip; LC = lip cavity.)

Within *P.* sect. Macrophyllae-Fasciculatae, although several species possess a concave labellum, very few develop a lip as deeply concave as that of the *P. crateriformis* complex. While the recently described *P. rikseniana* Mark Wilson & B.T. Larsen and *P. sabanillae* M.M. Jiménez & Vélez-Abarca of the *P. cardiostola–P. lilijae* complex and *Pleurothallis canaligera* Rchb.f. exhibit particularly concave lips, the deeply concave, saccate lip of the *P. crateriformis* complex is almost unique. This deeply concave lip is most obvious in *P. crateriformis* and *P. pyelophera*, as is apparent in the scanning electron micrograph (Fig. 14). Unlike in the majority of section Macrophyllae-Fasciculatae, where nectar-like liquid is produced on the epichile or mesochile of the lip and separately in the glenion, for example, in species of the *P. cardiostola–P. lilijae* group (Wilson et al. 2022), in the *P. crateriformis* complex the cavity of the lip is often filled with what is presumed to be nectar. Studies are underway at Colorado College to determine the sugar composition of this fluid using liquid chromatography–mass spectrometry (LC–MS) (Wilson & Brasuel, unpubd.) to confirm that it is nectar, as presumed.

**Figure 15.**
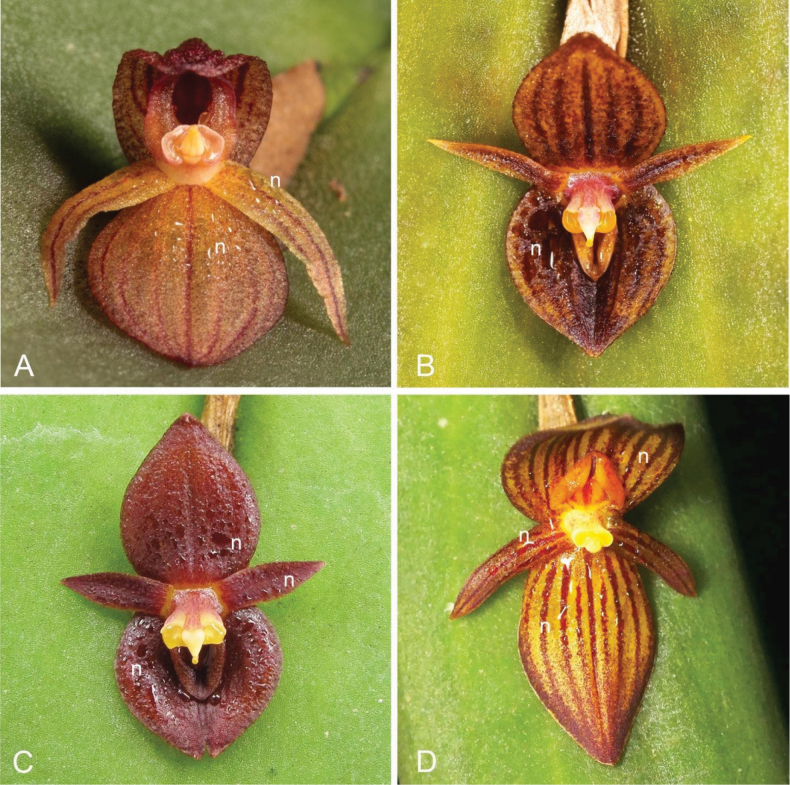
Extra-labellar nectar-like liquid on sepals or petals (**n**). **A**. *P. pyelophera* (*ex situ* collection of the Colorado College greenhouse, photo by M. Wilson); **B**. *P. equipedites* (*ex situ* collection, photo by Ron Parsons); **C**. *Pleurothallis* sp. (*ex situ* collection of Ecuagenera, photo by M. Wilson); **D**. *P. lynniana* (southeastern Ecuador, photo by Marco Jiménez).

In addition to the nectar-like liquid present within the lip cavity, some of these species, including *P. pyelophera*, *P. equipedites*, *P. lynniana*, and an undescribed species (Fig. 15), produce extra-labellar nectar-like secretions on the sepals or petals. In section Macrophyllae-Fasciculatae, this phenomenon appears to be unique to the *P. crateriformis* complex. Presuming that this liquid contains sugar and functions as nectar, we hypothesize that these extra-labellar secretions may act as supplementary attractants, guiding pollinators onto the flower and attracting them to the lip cavity, where additional nectar-like liquid is present.

The production of non-resupinate flowers, wherein the lip is uppermost, is by no means unique to the *P. crateriformis* complex; however, the majority of species in section Macrophyllae-Fasciculatae produce resupinate flowers (see Dressler 1981 for resupinate vs. non-resupinate). In *P. crateriformis*, *P. pyelophera*, and *P. phymatodea*, the flowers are mostly non-resupinate, and in *P. austrorientalis*, and *P. monteroae*, the flowers are sometimes non-resupinate. Why these species do not produce all non-resupinate flowers is unclear. However, species of Macrophyllae-Fasciculatae producing resupinate flowers almost never produce a non-resupinate flower. We hypothesize that, in species of the *P. crateriformis* complex that consistently produce non-resupinate flowers, the combination of the lip cavity filled with nectar-like liquid and the extra-labellar nectar-like liquid on the sepals or petals may lead to a different pollinator interaction compared with the majority of species in the section.

**Figure 16.**
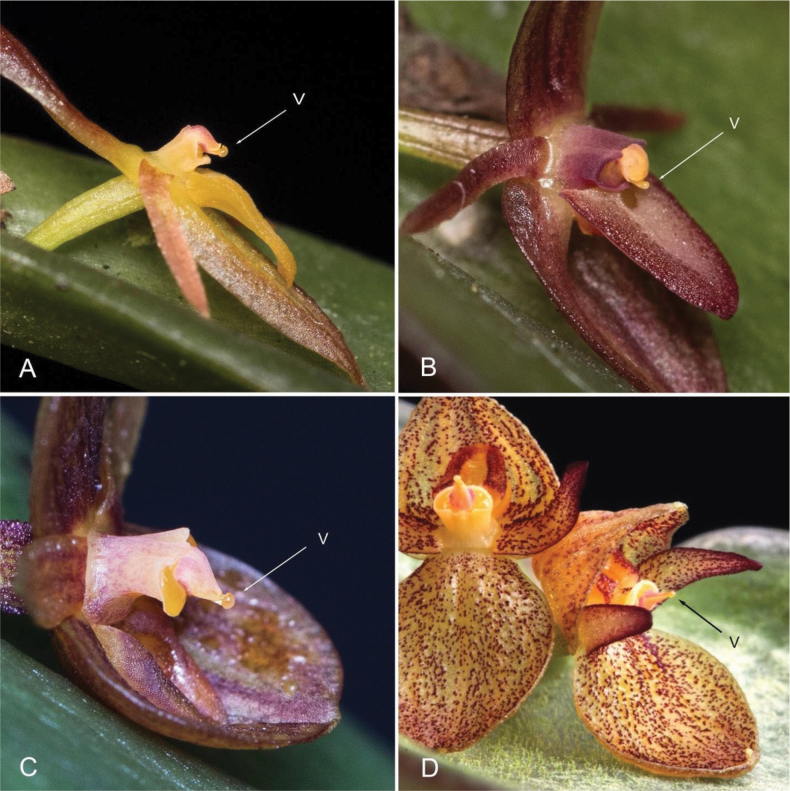
Viscidium (**v**) position in *Pleurothallis* sect. Macrophyllae-Fasciculatae. **A**. Typical member of *P.* sect. Macrophyllae-Fasciculatae, *Pleurothallis* sp. (photo by Kevin Holcomb); **B**. Typical member of *P.* sect. Macrophyllae-Fasciculatae, *Pleurothallis erythrium* (photo by Kevin Holcomb); **C**. Resupinate member of the *P. crateriformis* complex, *Pleurothallis equipedites* (photos by Kevin Holcomb); **D**. Non-resupinate member of the *P. crateriformis* complex, *Pleurothallis austrorientalis* (photo by Wiel Driessen).

**Figure 17.**
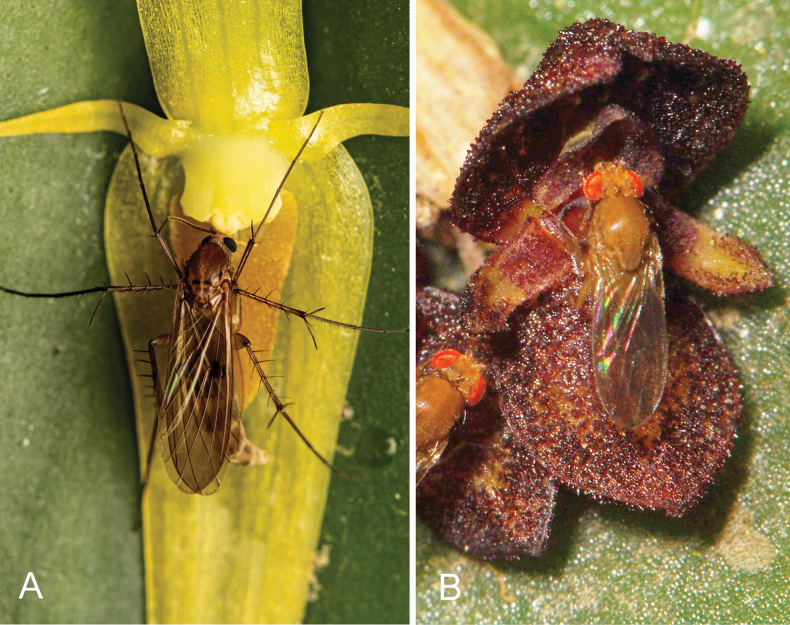
**A**. Resupinate flower of *Pleurothallis microcardia* with a putative pollinator (possibly Mycetophilidae) in a position leading to nototribic pollination (photo by Carlos Mesa Londoño). **B**. Non-resupinate flower of *Pleurothallis phymatodea* with a putative pollinator (possibly Drosophilidae) in a position leading to sternotribic pollination (photo by Francisco Tobar).

In the majority of species of *P.* sect. Macrophyllae-Fasciculatae, and possibly also in the resupinate species of the *P. crateriformis* complex, the lip morphology, together with the position of the glenion and the viscidium (Fig. 16A, B, C), results in dorsal pollinarium deposition on the head of the pollinator (Figs 5, 17A). In contrast, we hypothesize that in the non-resupinate species of the *P. crateriformis* complex, the unusual position of the viscidium, located at the distal end of the pollinarium and held upward by the rostellum rather than directed forward (Fig. 16D), results in ventral pollinarium attachment on the pollinator’s thorax. A shift from nototribic to sternotribic pollination may thus have occurred in this complex.

We further hypothesize that the pollinating insects, approaching the flower in response to visual and olfactory cues, are attracted onto the floral surface by extra-labellar nectar present on the dorsal sepal or petals and then advance over the anther to reach nectar within the cavity of the non-resupinate lip. The cucullate synsepal, as observed in *P. crateriformis* and *P. pyelophera* (Fig. 2), likely restricts access to the nectar in the lip cavity from above; consequently, even if the insect approached from above, the position of the viscidium would impede pollinarium acquisition. The observed interaction of a drosophilid with non-resupinate flowers of *P. phymatodea* (Fig. 17B) provides preliminary support for the hypothesized pollination mechanism.

Finally, an interesting observation is the marked morphological similarity between the non-resupinate flowers of the *P. crateriformis* complex from Ecuador and Peru, such as *P. phymatodea* (Fig. 4A), and those of the Mesoamerican species (Pupulin and Zúñiga 2007), such as *P. excavata* (Fig. 4B). This could suggest either convergent evolution to a similar pollination mechanism or phylogenetic relatedness between the two groups. Convergent evolution is common in Orchidaceae and in Pleurothallidinae in particular (Karremans 2016) and has resulted in confusion in morphologically based taxonomies (for example, Wilson et al. 2017). In this case, we hypothesize that there has been convergent evolution between the two groups to a similar pollination mechanism entailing a shift in pollinarium site attachment (Dressler 1968), from attachment on the head of the pollinator (nototribic) (Fig. 5) to attachment on the underside of the thorax (sternotribic). Support for this hypothesis includes the wide geographic (~1700 km) separation between the two groups and observations and data suggesting that the two groups are not closely related. The Mesoamerican species possess a single apical stigmatic surface typical of section *Pleurothallis*, rather than the paired apical stigmatic surfaces of section Macrophyllae-Fasciculatae, and exhibit distinctly different vegetative morphology. Further, preliminary ITS and *matK* phylogenetic data also suggest that the Mesoamerican species represent a clade in section *Pleurothallis* rather than section Macrophyllae-Fasciculatae (Wilson et al. 2013). If ongoing phylogenomic studies (Arias, Eserman & Wilson, unpubd.) support these phylogenetic results, this would represent the most dramatic case of convergent evolution in *Pleurothallis*.

Ongoing studies related to pollination biology in the *Pleurothallis crateriformis* complex include analyses of the sugar composition of the nectar-like liquid within the crateriform lip using liquid chromatography–mass spectrometry (LC–MS) and analyses of floral volatiles using gas chromatography–mass spectrometry (GC–MS) (Wilson & Brasuel, unpubd.).

## Supplementary Material

XML Treatment for Pleurothallis monteroae

XML Treatment for Pleurothallis austrorientalis

XML Treatment for Pleurothallis crateriformis

XML Treatment for Pleurothallis equipedites

XML Treatment for Pleurothallis nipterophylla

XML Treatment for Pleurothallis phymatodea

XML Treatment for Pleurothallis pyelophera

## Additional information

### Use of AI

No use of AI was reported.

### Author ORCIDs

Marco M. Jiménez https://orcid.org/0000-0002-9502-5651

Gabriel A. Iturralde https://orcid.org/0000-0003-2456-0929

Nadia Lapo-González https://orcid.org/0009-0001-8863-1557

Henry X. Garzón-Suárez https://orcid.org/0000-0003-4049-1652

Raven Ward https://orcid.org/0000-0003-3245-4931

Mark Wilson https://orcid.org/0000-0003-3044-7471

### Data availability

All of the data that support the findings of this study are available in the main text or Supplementary Information.

## Supplementary materials

Supplementary material 1 iNaturalist record of *Pleurothallis monteroae* Data type docx https://binary.pensoft.net/file/1535896This dataset is made available under the Open Database License (http://opendatacommons.org/licenses/odbl/1.0/). The Open Database License (ODbL) is a license agreement intended to allow users to freely share, modify, and use this Dataset while maintaining this same freedom for others, provided that the original source and author(s) are credited. Marco M. Jiménez, Gabriel A. Iturralde, Nadia Lapo-González, Henry X. Garzón-Suárez, Gerhard Vierling, Raven Ward, Mark Wilson

Supplementary material 2 iNaturalist record of *Pleurothallis nipterophylla* Data type docx https://binary.pensoft.net/file/1535897This dataset is made available under the Open Database License (http://opendatacommons.org/licenses/odbl/1.0/). The Open Database License (ODbL) is a license agreement intended to allow users to freely share, modify, and use this Dataset while maintaining this same freedom for others, provided that the original source and author(s) are credited. Marco M. Jiménez, Gabriel A. Iturralde, Nadia Lapo-González, Henry X. Garzón-Suárez, Gerhard Vierling, Raven Ward, Mark Wilson

Supplementary material 3 iNaturalist record of *Pleurothallis phymatodea* Data type docx https://binary.pensoft.net/file/1535898This dataset is made available under the Open Database License (http://opendatacommons.org/licenses/odbl/1.0/). The Open Database License (ODbL) is a license agreement intended to allow users to freely share, modify, and use this Dataset while maintaining this same freedom for others, provided that the original source and author(s) are credited. Marco M. Jiménez, Gabriel A. Iturralde, Nadia Lapo-González, Henry X. Garzón-Suárez, Gerhard Vierling, Raven Ward, Mark Wilson

## References

[B1] Bachman S, Moat J, Hill AW, de la Torre J, Scott B (2011) Supporting Red List threat assessments with GeoCAT: Geospatial Conservation Assessment Tool. ZooKeys 150: 117–126. 10.3897/zookeys.150.2109 PMC3234434 22207809

[B2] Beck E, Makeschin F, Haubrich F, Richter M, Bendix J, Valerezo C (2018) Chapter 1. The Ecosystem (Reserva Biológica San Francisco). In: Beck E, Bendix J, Kottke I, Makeschin F, Mosandl R (Eds) Gradients in a Tropical Mountain Ecosystem of Ecuador. Ecological Studies, Vol. 198. Analysis and Synthesis. Springer-Verlag Berlin Heidelberg, Heidelberg, 1–12. 10.1007/978-3-540-73526-7_1

[B3] Beentje H (2016) The Kew Plant Glossary: an illustrated dictionary of plant terms, Second Edition. Kew Publishing, Royal Botanical Gardens, Richmond, 184 pp.

[B4] Dodson CH (1962) The importance of pollination in the evolution of the orchids of tropical America. American Orchid Society Bulletin 31: 525–534.

[B5] Dressler RL (1968) Pollination by Euglossine bees. Evolution 22(1): 202–210. 10.1111/j.1558-5646.1968.tb03463.x 28564982

[B6] Dressler RL (1981) The Orchids: Natural History and Classification. The Smithsonian Institution, United States of America, 326 pp.

[B7] Duque O (1993) Polinización en *Pleurothallis*. Orquideología 19: 55–69.

[B8] Duque-Buitrago CA, Alzate-Quintero NF, Tupac Otero J (2014) Nocturnal pollinatIon by fungus gnats of the colombian endemic species, *Pleurothallis marthae* (orchidaceae: Pleurothallidinae). Lankesteriana 13(3): 407–417. 10.15517/lank.v13i3.14429

[B9] ESRI (2024) ArcGIS Desktop: Release 10.3. Environmental Systems Research Institute, Redlands, California.

[B10] Holcomb KW (2023) *Pleurothallis equipedites* K.W.Holcomb, sp. nov. Pleurothallidinae 2(6): 1.

[B11] IUCN (2024) IUCN Standards and Petitions Committee 2024. Guidelines for Using the IUCN Red List Categories and Criteria. Version 16. Prepared by the Standards and Petitions Committee. https://www.iucnredlist.org/resources/redlistguidelines [accessed 04.11.2025]

[B12] Karremans AP (2016) Genera Pleurothallidinarum: An updated phylogenetic overview of Pleurothallidinae. Lankesteriana 16(2): 219–214. 10.15517/lank.v16i2.26008

[B13] Karremans AP, Díaz-Morales M (2019) The Pleurothallidinae: Extremely high speciation driven by pollinator adaptation. In: Pridgeon AM, Arosemena AR (Eds) Proceedings of the 22^nd^ World Orchid Conference, Vol. 1. (363–388). Guayaquil, Ecuador: Asociación Ecuatoriana de Orquideología.

[B14] Kleemann J, Zamora C, Villacis-Chiluisa AB, Cuenca P, Koo H, Noh JK, Fürst C, Thiel M (2022) Deforestation in continental Ecuador with a focus on protected areas. Land 11(2): 268. 10.3390/land11020268

[B15] Luer CA (1976) Miscellaneous species of *Pleurothallis*. Selbyana 3(1/2): 38–201.

[B16] Luer CA (2005) Icones Pleurothallidinarum XXVII: *Dryadella* and *Acronia* section *Macrophyllae-Fasciculatae*. In: Monographs in Systematic Botany from the Missouri Botanical Garden 103: 1–311.

[B17] Luer CA (2006) Icones Pleurothallidinarum XXVIII: A reconsideration of *Masdevallia*. Systematics of *Specklinia* and vegetatively similar taxa (Orchidaceae). In: Monographs in Systematic Botany from the Missouri Botanical Garden 105: 1–281.

[B18] Mena-Quintana FN, Álvarez W, Franco W, Moncayo L, Tipán M, Ayala J (2025) Land degraded by gold mining in the Ecuadorian Amazon: A proposal for boosting eco­system restoration through induced revegetation. Forests 16(2): 372. 10.3390/f16020372

[B19] Mestanza-Ramón C, Cuenca-Cumbicus J, D’orio G, Flores-Toala J, Segovia-Cáceres S, Bonilla-Bonilla A, Straface S (2022) Gold mining in the Amazon region of Ecuador: History and a review of its socio-environmental impacts. Land 11(2): 221. 10.3390/land11020221

[B20] Ministerio del Ambiente del Ecuador (2013) Sistema de Clasificación de los Ecosistemas del Ecuador Continental. Subsecretaría de Patrimonio Natural, Quito, 232 pp.

[B21] Noh JK, Echeverria C, Gaona G, Kleemann J, Koo H, Fürst C, Cuenca P (2022) Forest ecosystem fragmentation in Ecuador: Challenges for sustainable land use in the Tropical Andean. Land 11(2): 287. 10.3390/land11020287

[B22] Peck MR, Desselas M, Bonilla‐Bedoya S, Redín G, Durango‐Cordero J (2024) The conflict between Rights of Nature and mining in Ecuador: Implications of the Los Cedros Cloud Forest case for biodiversity conservation. People and Nature 6(3): 1096–1115. 10.1002/pan3.10615

[B23] POWO (2025) Plants of the World Online. Facilitated by the Royal Botanic Gardens, Kew. https://powo.science.kew.org/ [accessed 04.10.2025]

[B24] Pupulin F, Zúñiga JD (2007, September) The upside-down *Pleurothallis* (Orchidaceae) of Mesoamerica, with a new species from Costa Rica. Lindleyana, 690–695. [Orchids Magazine]

[B25] Pupulin F, Díaz-Morales M, Aguilar J, Fernández M (2017) Two new species of *Pleurothallis* (Orchidaceae: Pleurothallidinae) allied to *P. cardiothallis*, with a note on flower activity. Lankesteriana 17(2): 329–356. 10.15517/lank.v17i2.30272

[B26] Pupulin F, Aguilar J, Belfort-Oconitrillo N, Díaz-Morales M, Bogarín D (2021) Florae Costaricensis subtribu Pleurothallidinis (Orchidaceae) prodromus II. Systematics of the *Pleurothallis cardiothallis* and *P. phyllocardia* groups, and other related groups of *Pleurothallis* with large vegetative habit. Harvard Papers in Botany 26(1): 203–295. 10.3100/hpib.v26iss1.2021.n14

[B27] Quizhpe W, Aguirre ZM, Cabrera O, Delgado TE (2002) Los páramos del Parque Nacional Podocarpus. In: Aguirre ZM, Madsen JE, Cotton E, Balslev H (Eds) Botánica Austroecuatoriana. Estudios sobre los recursos vegetales en las provincias de El Oro, Loja y Zamora-Chinchipe. Ediciones Abya Yala, Quito, 79–90.

[B28] Revatta-Bustos F, Edquén JD, Arista JP, Yrigoín E, Rivera López RY, Wilson M, Enco M, Edquen K, Leiva-Espinoza S, Oliva-Cruz M, Salazar GA (2025) A new species of *Pleurothallis* in the *P. cardiostola*-*P. lilijae* complex of section *Macrophyllae-Fasciculatae* (Orchidaceae, Pleurothallidinae) from Ecuador and Peru. PhytoKeys 262: 279–292. 10.3897/phytokeys.262.157111 PMC12449696 40979458

[B29] Rojas-Alvarado G, Karremans A (2024) A typological and morphological analysis of the Pleurothallidinae (Orchidaceae) inflorescences. Botanical Review 90(3): 221–250. 10.1007/s12229-024-09303-6

[B30] Roy BA, Zorrilla M, Endara L, Thomas DC, Vandegrift R, Rubenstein JM, Policha T, Ríos-Touma B, Read M (2018) New mining concessions could severely decrease biodiversity and ecosystem services in Ecuador. Tropical Conservation Science 11: 1940082918780427. 10.1177/1940082918780427

[B31] Schweinfurth C (1951) Orchidaceae Peruvianae VIII. Botanical Museum Leaflets, Harvard University 15(3): 79–110. 10.5962/p.168474

[B32] Tapia-Armijos MF, Homeir J, Espinosa CI, Leuschner C, de la Cruz M (2015) Deforestation and forest fragmentation in South Ecuador since the 1970s – Losing a hotspot of biodiversity. PLoS ONE 10(11): e0142359. 10.1371/journal.pone.0142359 PMC4557835 26332681

[B33] Thiers BM (2025) Updated continuously. Index Herbariorum. https://sweetgum.nybg.org/science/ih/ [accessed 04.11.2025]

[B34] Wilson M, Pupulin F, Archila-Morales F, Damon A, Solano-Gomez R (2013) A newly recognized clade of *Pleurothallis* with Mesoamerican distribution. Lankesteriana 13(1–2): 138. 10.15517/lank.v0i0.11567 [Poster presented at IV Conferencia Científica de Orquideas Andinas, Guayaquil, Ecuador, Nov 2012.]

[B35] Wilson M, Baquero L, Dupree K, Jiménez M, LeBlanc C, Merino G, Portilla J, Salas Guerrero M, Tobar Suarez F, Werner J (2016) Three new species of *Pleurothallis* (Pleurothallidinae; Orchidaceae) in subsection *Macrophyllae*-*Fasciculatae* from northern South America. Lankesteriana 16: 349–366. 10.15517/lank.v16i3.27314

[B36] Wilson M, Frank GS, Jost L, Pridgeon A, Vieira-Uribe S, Karremans A (2017) Phylogenetic analysis of *Andinia* (Orchidaceae: Pleurothallidinae) and a systematic re-circumscription of the genus. Phytotaxa 295(2): 101–131. 10.11646/phytotaxa.295.2.1

[B37] Wilson M, Zhao K, Hampson H, Frank G, Romelroux K, Jiménez MM, Tobar F, Larsen B, Perez A (2018) A new species of *Pleurothallis* (Orchidaceae: Pleurothallidinae) in subsection *Macrophyllae-Fasciculatae* with a unique, highly reduced, morphologically distinct labellum. Lankesteriana 18: 217–230. 10.15517/lank.v18i3.35495

[B38] Wilson M, Larsen B, Moreno JS, Ward R, Riksen JAG, Pina L, Sierra-Ariza MA, Jiménez MM, Rincón-Gonzalez M, Galindo-Tarazona R, Garzón Suárez H, Haelterman D (2022) New species of *Pleurothallis* (Orchidaceae: Pleurothallidinae), a new country record, and labellar morphology in the *P. cardiostola-P. lilijae* complex of subsection *Macrophyllae-Fasciculatae*. Harvard Papers in Botany 27(2): 187–220. 10.3100/hpib.v27iss2.2022.n10

