# Supplementary material for: The Pleurothallis crateriformis complex (Orchidaceae): undescribed diversity and pollination biology of a newly recognized species group from Ecuador and Peru
Source: PhytoKeys. 2026 Feb 9;270:325–53. doi: 10.3897/phytokeys.270.175070 (PMC12910285; doi:10.3897/phytokeys.270.175070)

**Supplementary material 1:** iNaturalist record of *Pleurothallis monteroae*.


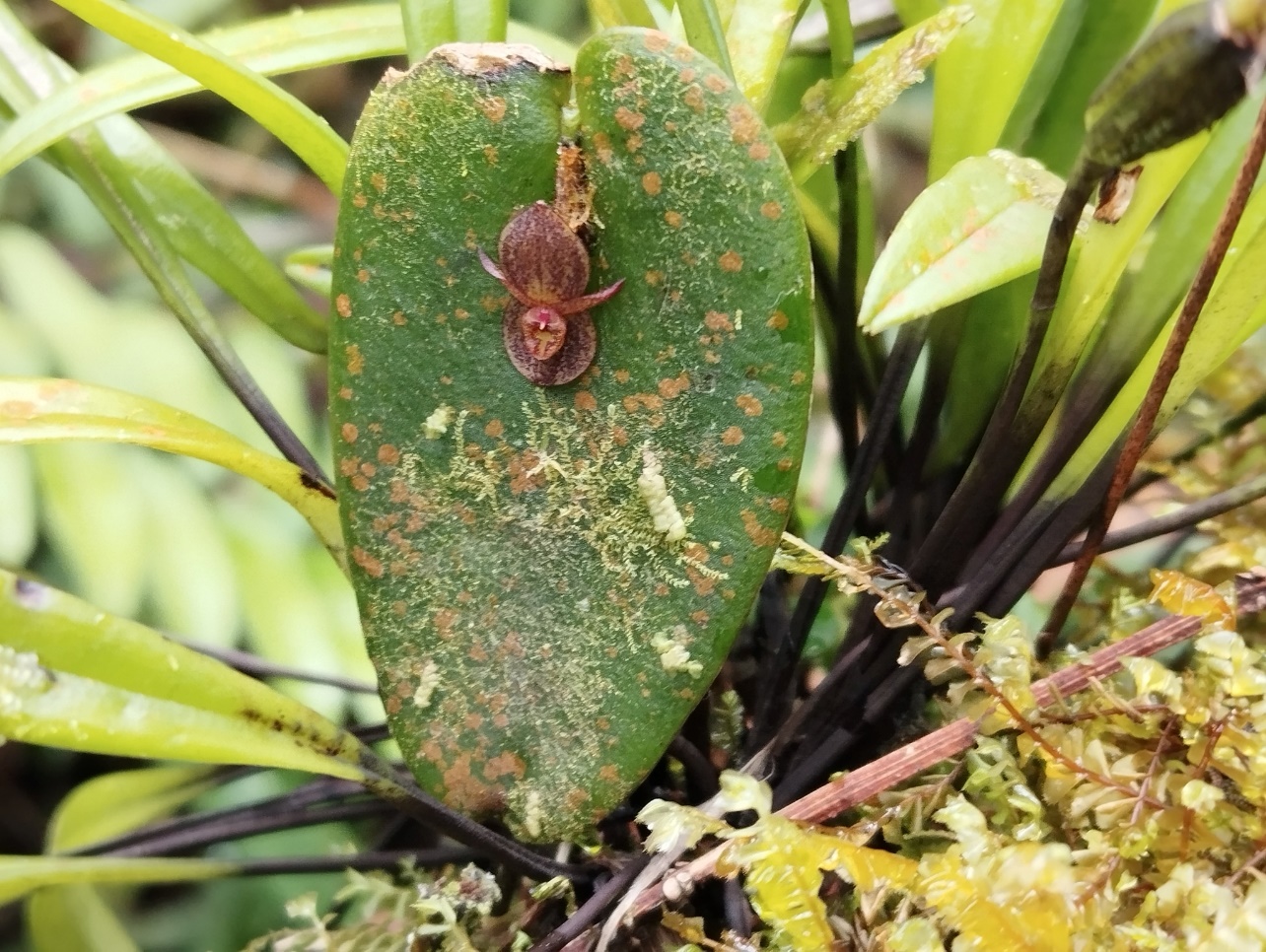

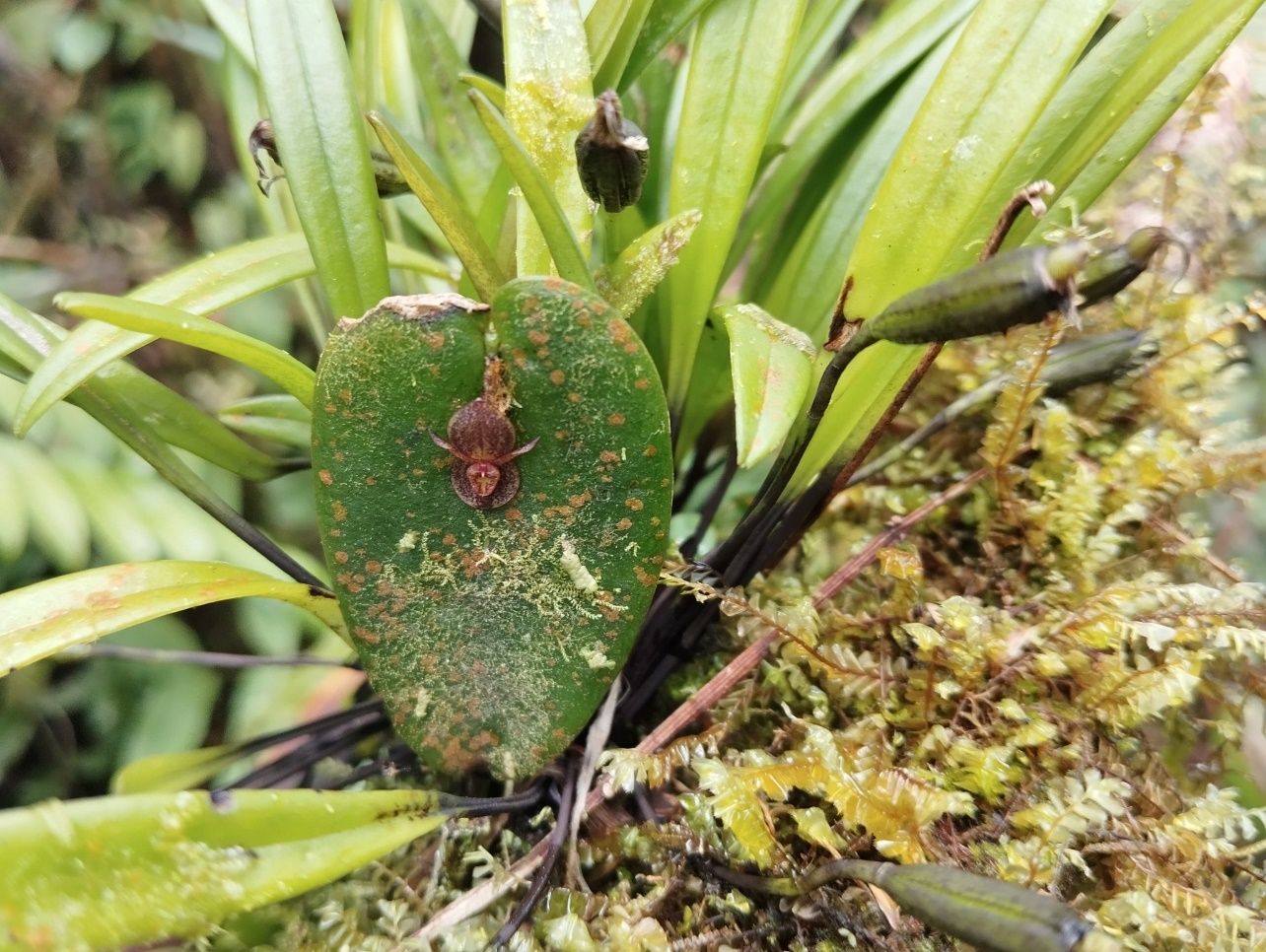
Name of record: *Pleurothallis* Section Macrophyllae-fasciculatae. Record by: Paola Ordóñez Montero. iNaturalist user: pao_montero. Date observed: 18 MAR 2025. Date published: 19 MAR 2025. General locality: Nangaritza, EC-ZC, EC. Latitude: -4.6056212. Longitude: -78.87687105. Accuracy: Not recorded. Geoprivacy: Open. Licensed under CC BY‑NC 4.0.
URL: <https://www.inaturalist.org/observations/265967102>.


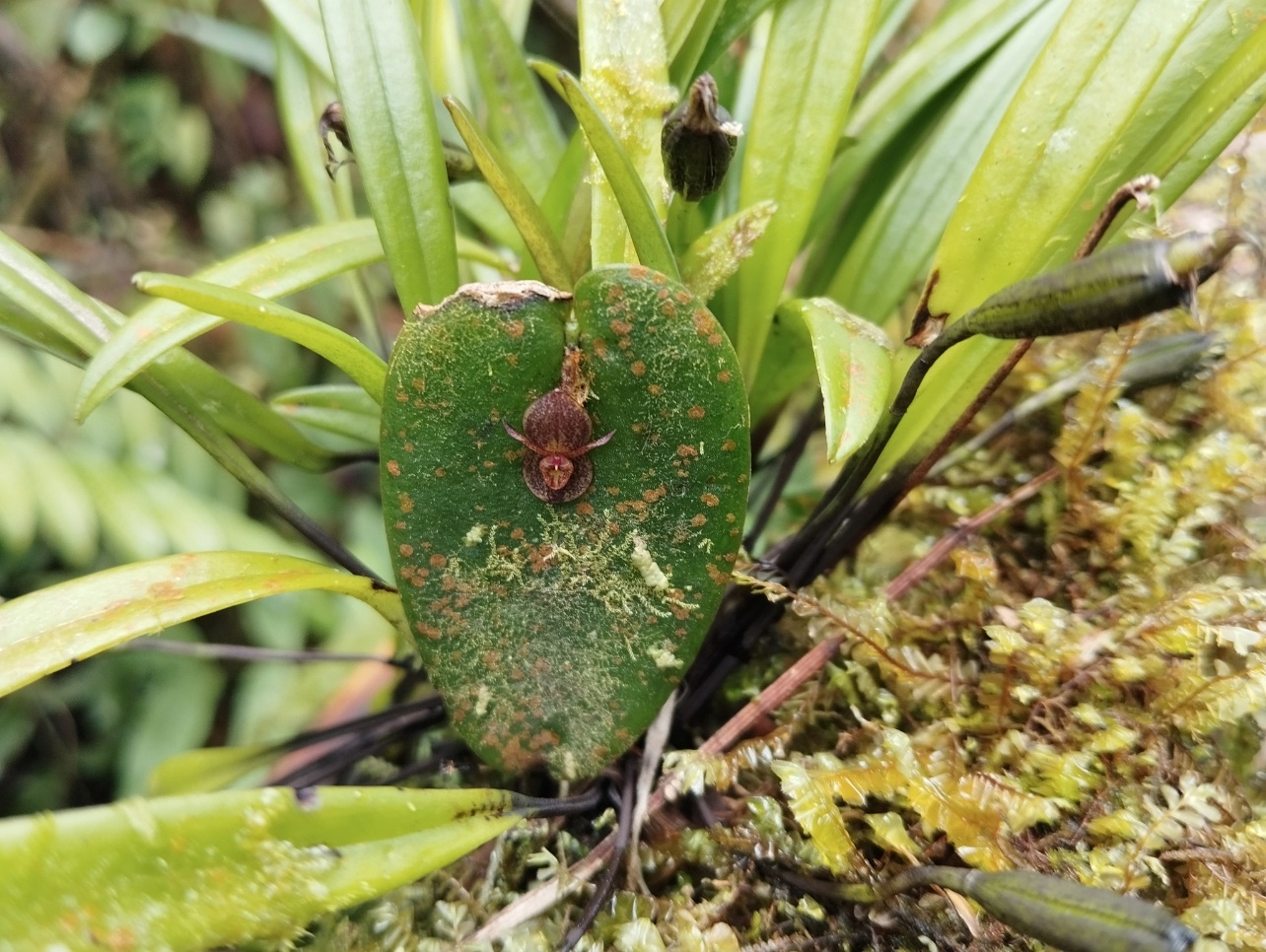

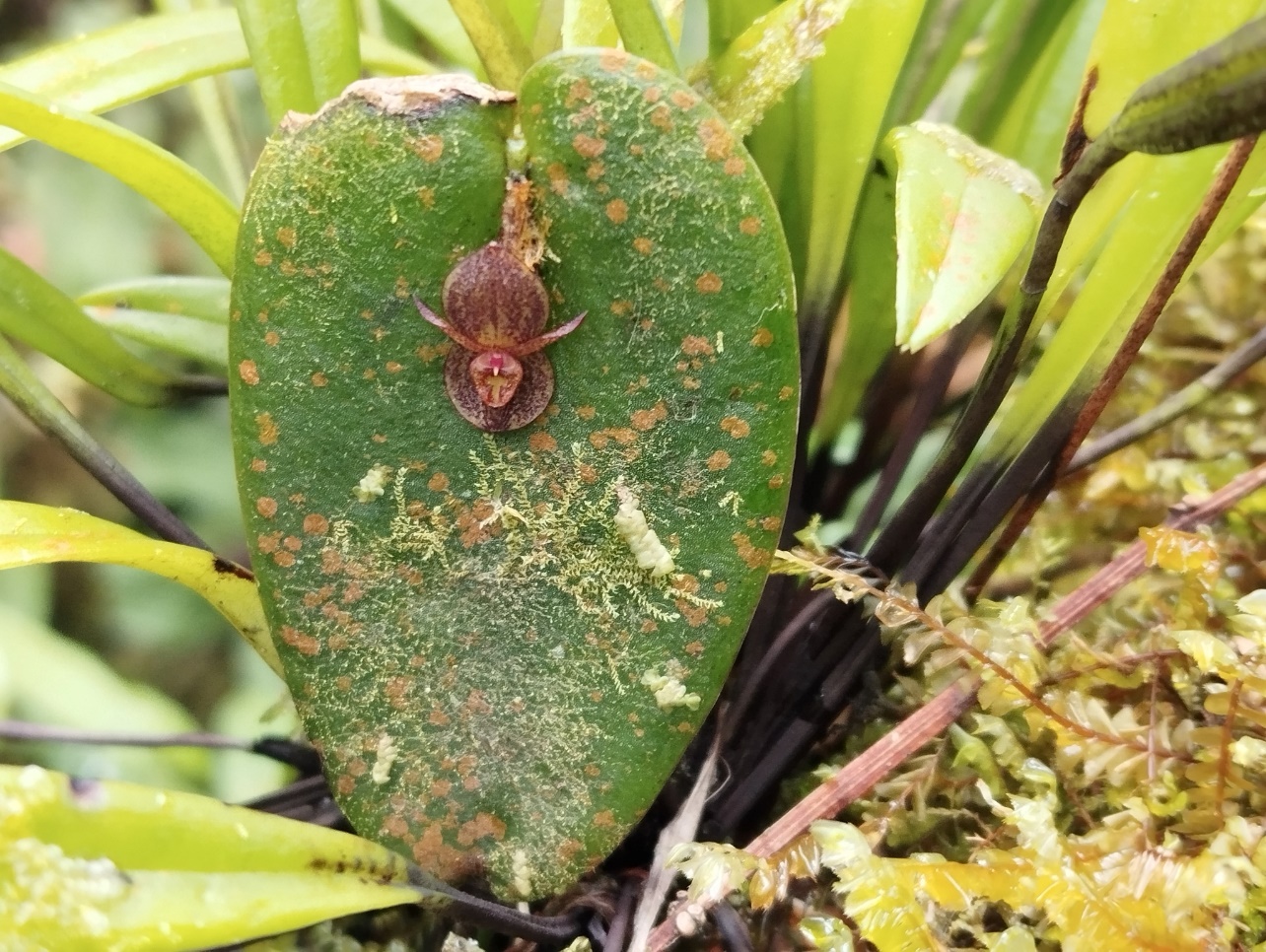


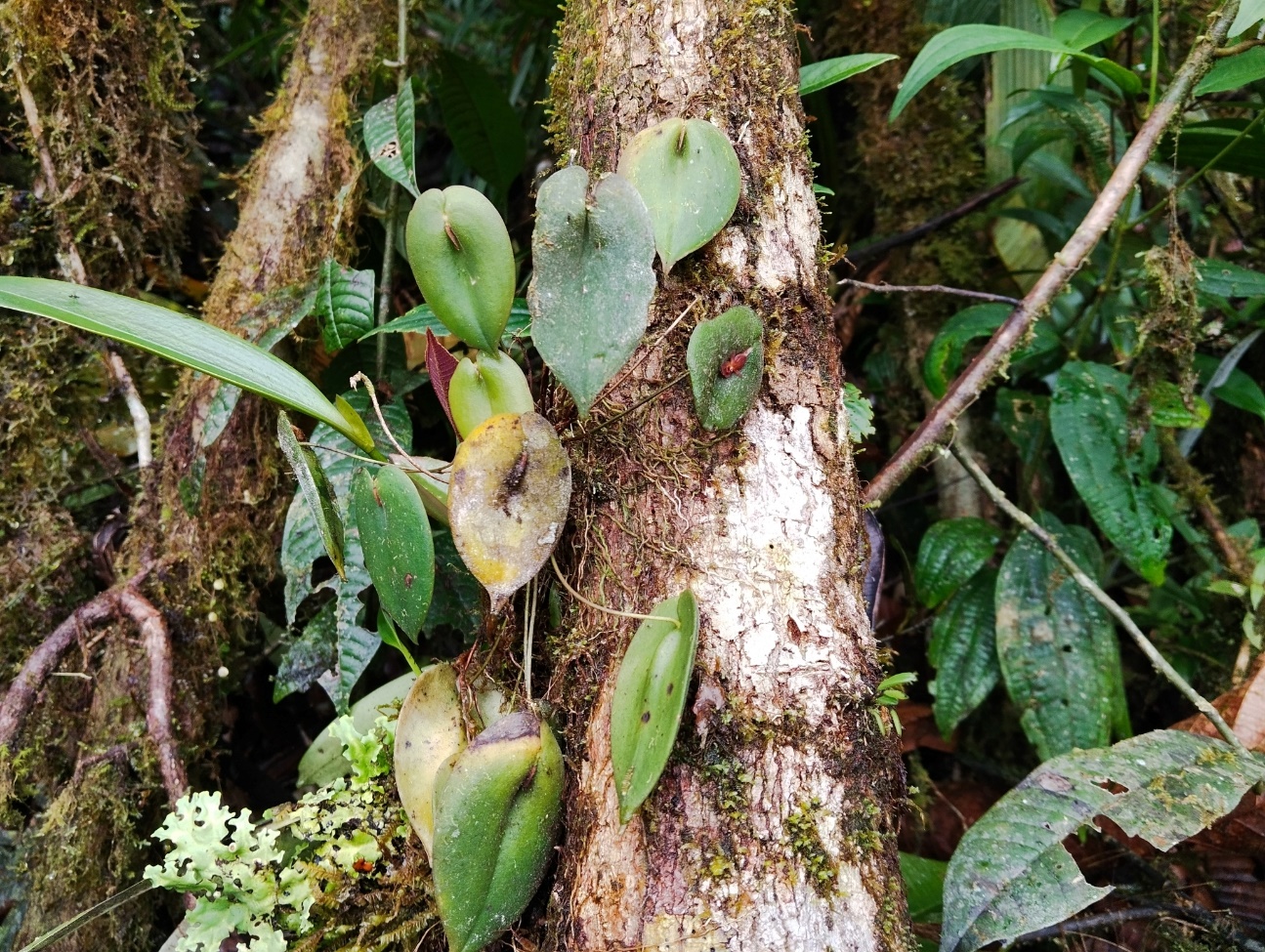

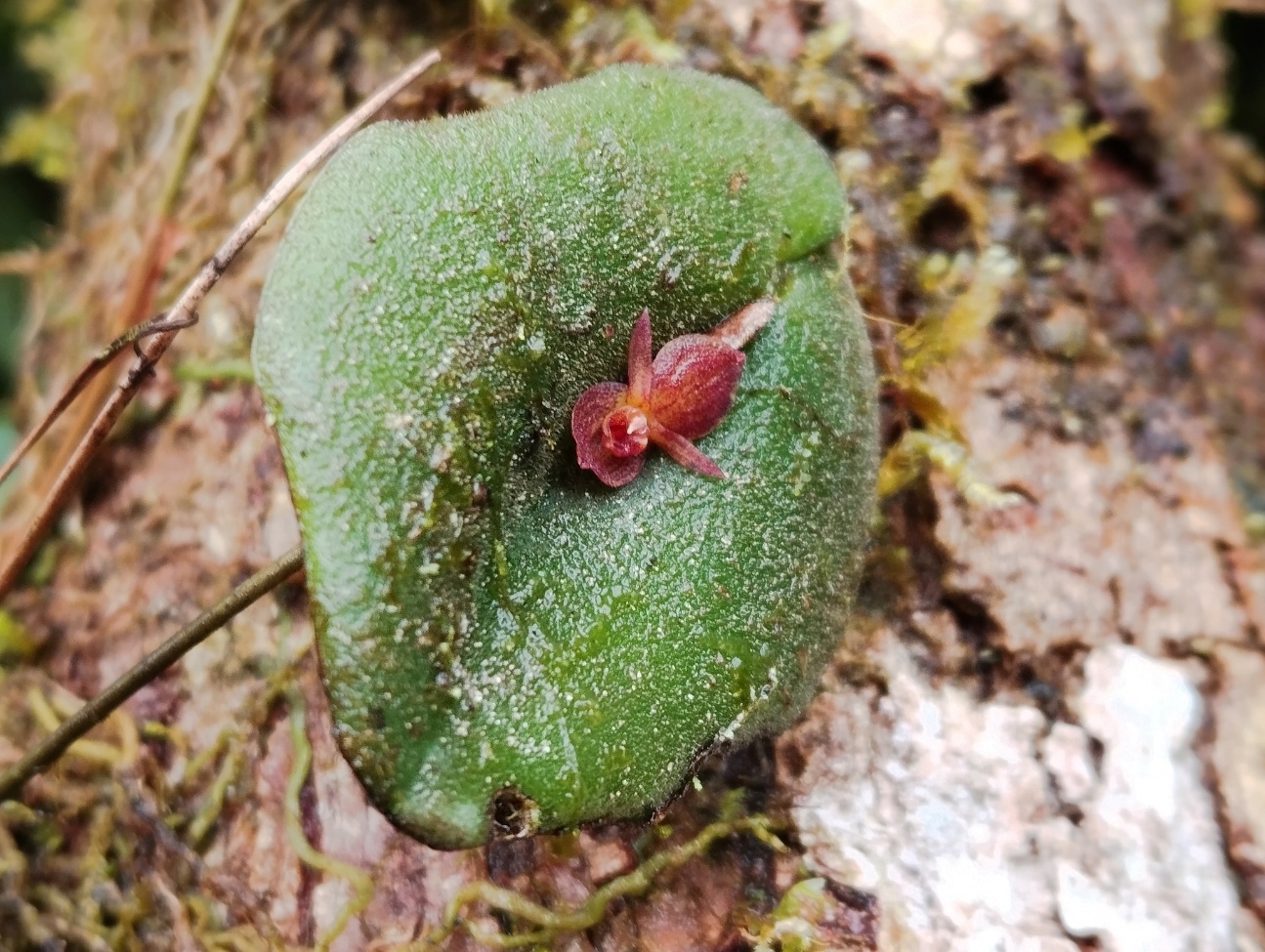
Name of record: *Pleurothallis*. Record by: Paola Ordóñez Montero. iNaturalist user: pao_montero. Date observed: 24 APR 2024. Date published: 24 APR 2024. General locality: Nangaritza, EC-ZC, EC. Latitude: -4.60425874. Longitude: -78.88073162. Licensed under CC BY‑NC 4.0.
URL: <https://www.inaturalist.org/observations/209190380>.


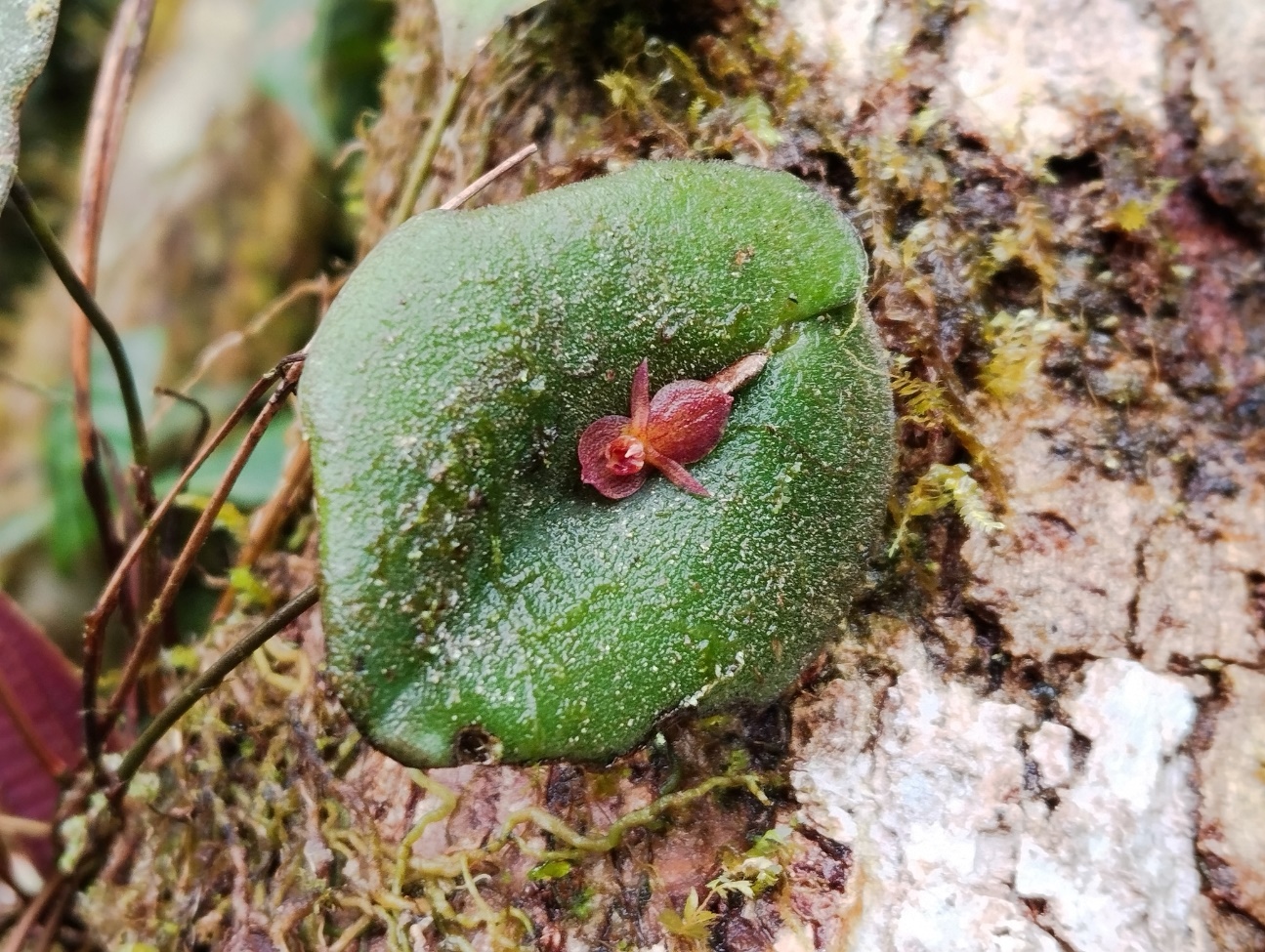

Supplement: Supplementary material 1 — iNaturalist record of Pleurothallis monteroae [file phytokeys-270-325_article-175070__-s001.docx]
