# Supplementary material for: The Pleurothallis crateriformis complex (Orchidaceae): undescribed diversity and pollination biology of a newly recognized species group from Ecuador and Peru
Source: PhytoKeys. 2026 Feb 9;270:325–53. doi: 10.3897/phytokeys.270.175070 (PMC12910285; doi:10.3897/phytokeys.270.175070)

**Supplementary material 2:** iNaturalist record of *Pleurothallis nipterophylla*.


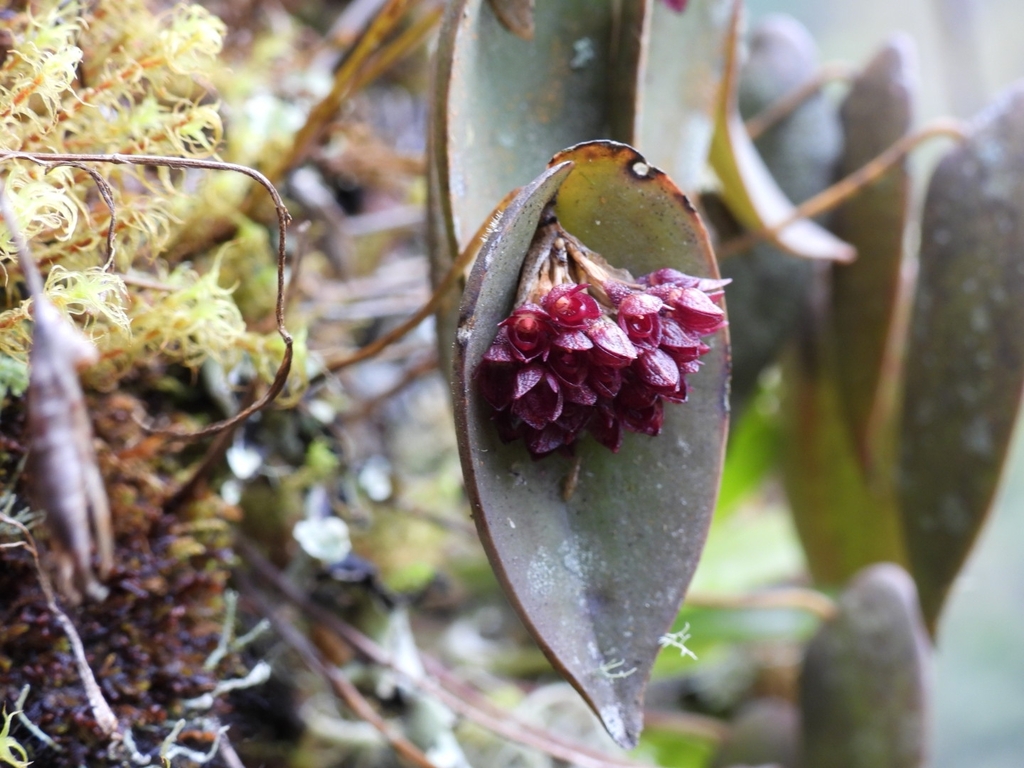

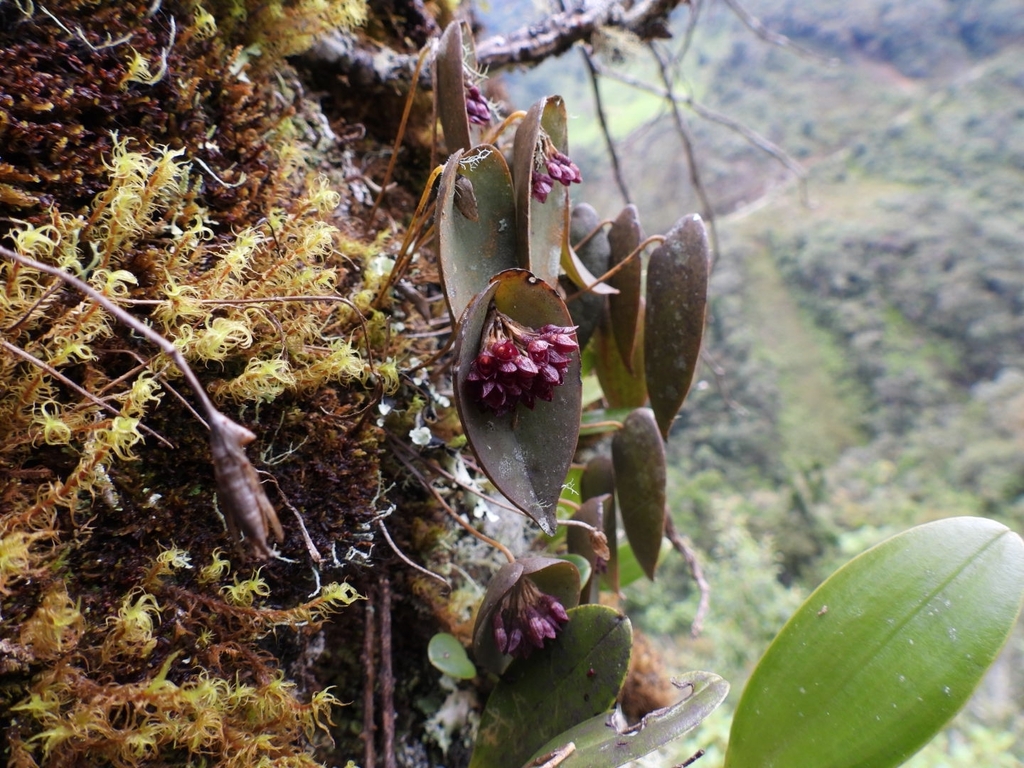
Name of record: *Pleurothallis nipterophylla*. Record by: Luis Salagaje. iNaturalist user: luissalagaje. Date observed: 20 FEB 2025. Date published: 20 FEB 2025. General locality: Gualaquiza, EC-MS, EC. Latitude: -3.3280983333. Longitude: -78.8468933333. Accuracy: 1 m. Geoprivacy: Open. Licensed under CC BY‑NC 4.0.
URL: <https://www.inaturalist.org/observations/262465969>.


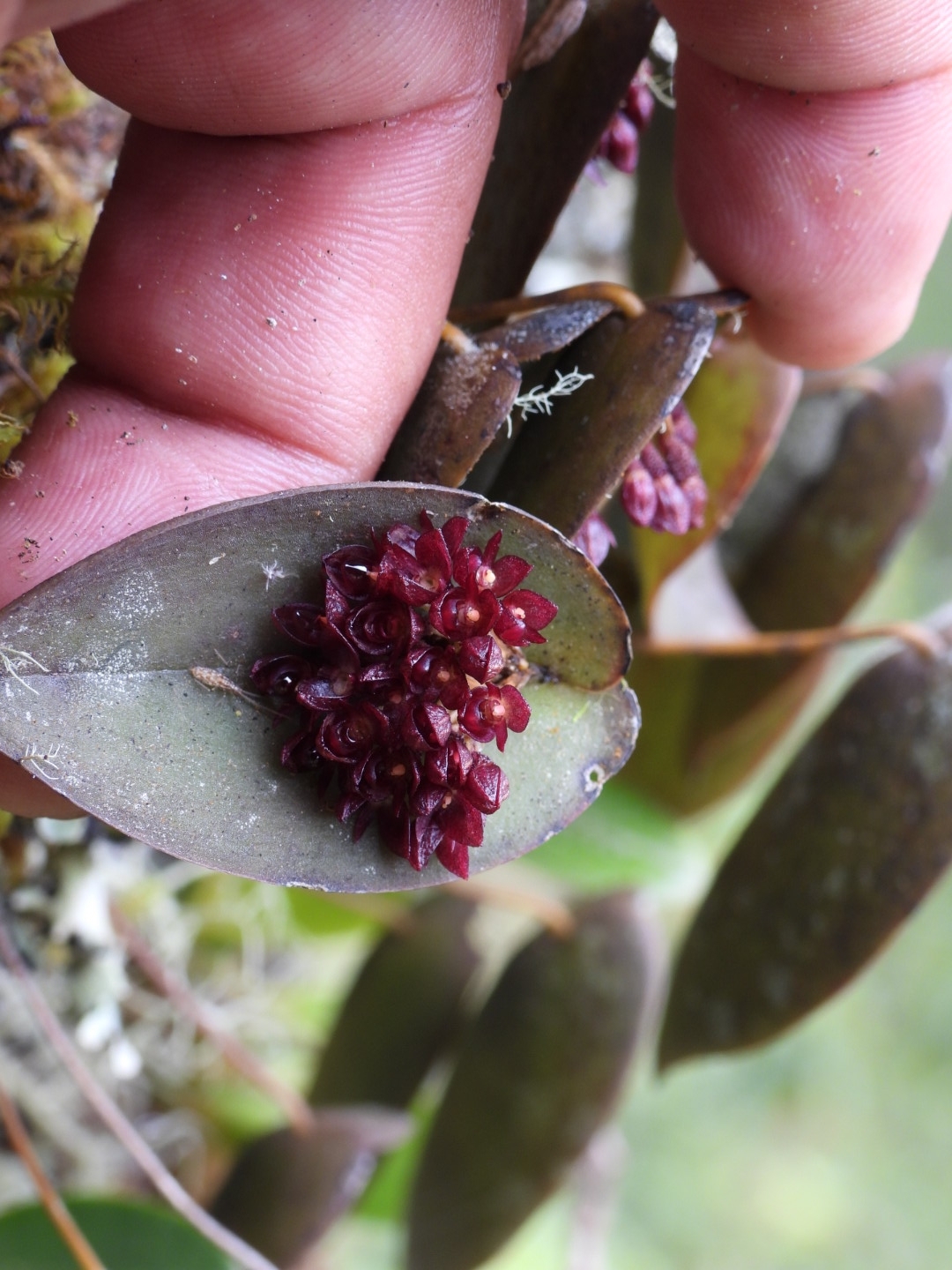


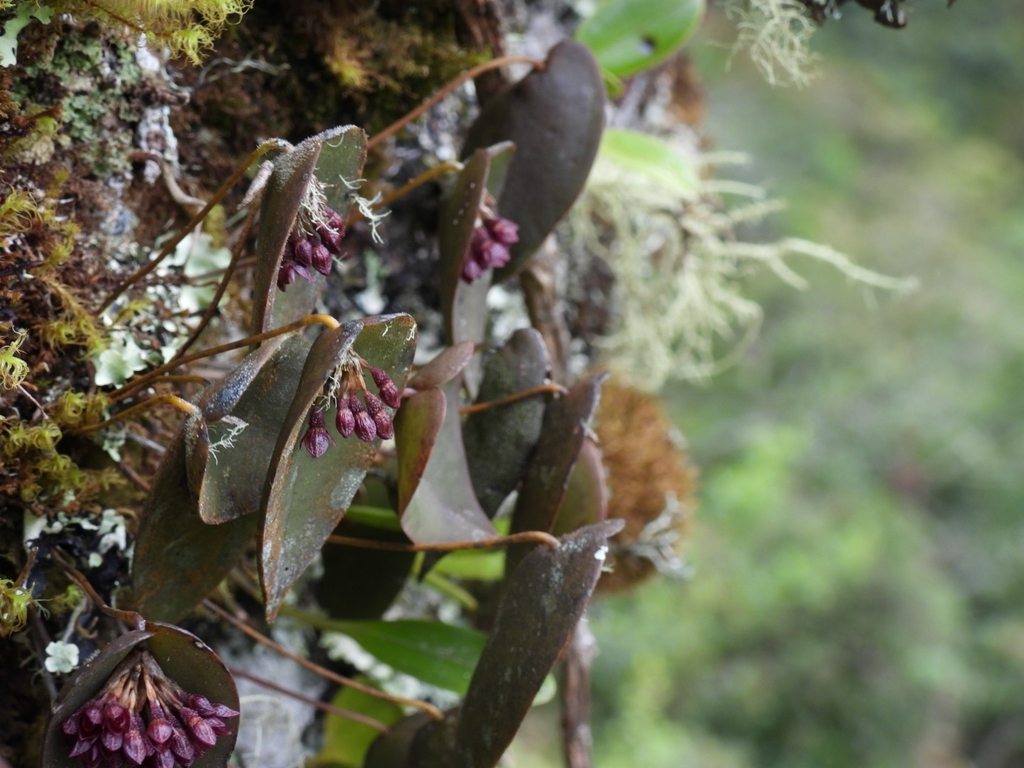

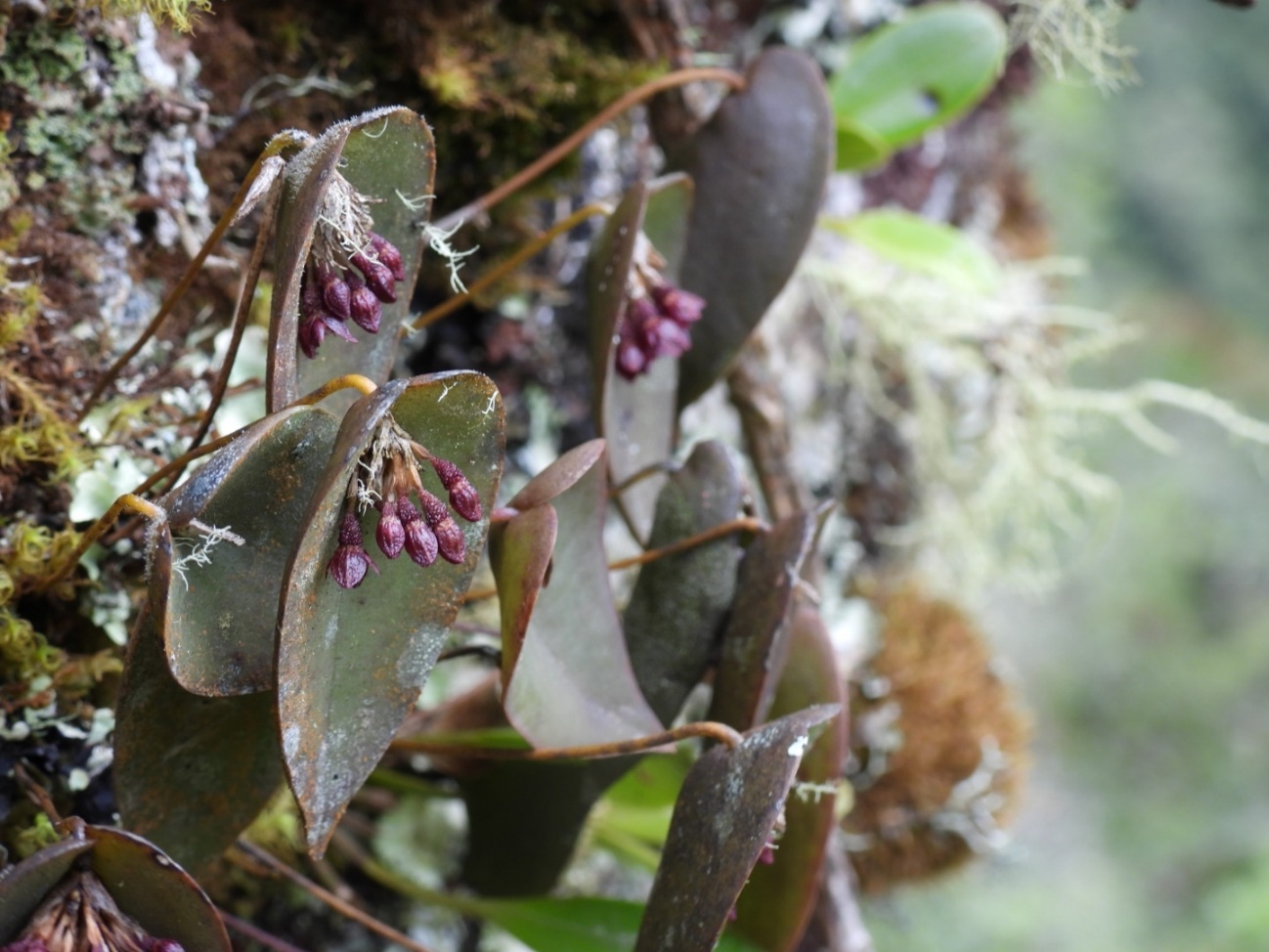

Supplement: Supplementary material 2 — iNaturalist record of Pleurothallis nipterophylla [file phytokeys-270-325_article-175070__-s002.docx]
