# Supplementary material for: The Pleurothallis crateriformis complex (Orchidaceae): undescribed diversity and pollination biology of a newly recognized species group from Ecuador and Peru
Source: PhytoKeys. 2026 Feb 9;270:325–53. doi: 10.3897/phytokeys.270.175070 (PMC12910285; doi:10.3897/phytokeys.270.175070)

**Supplementary material 3:** iNaturalist record of *Pleurothallis phymatodea*.


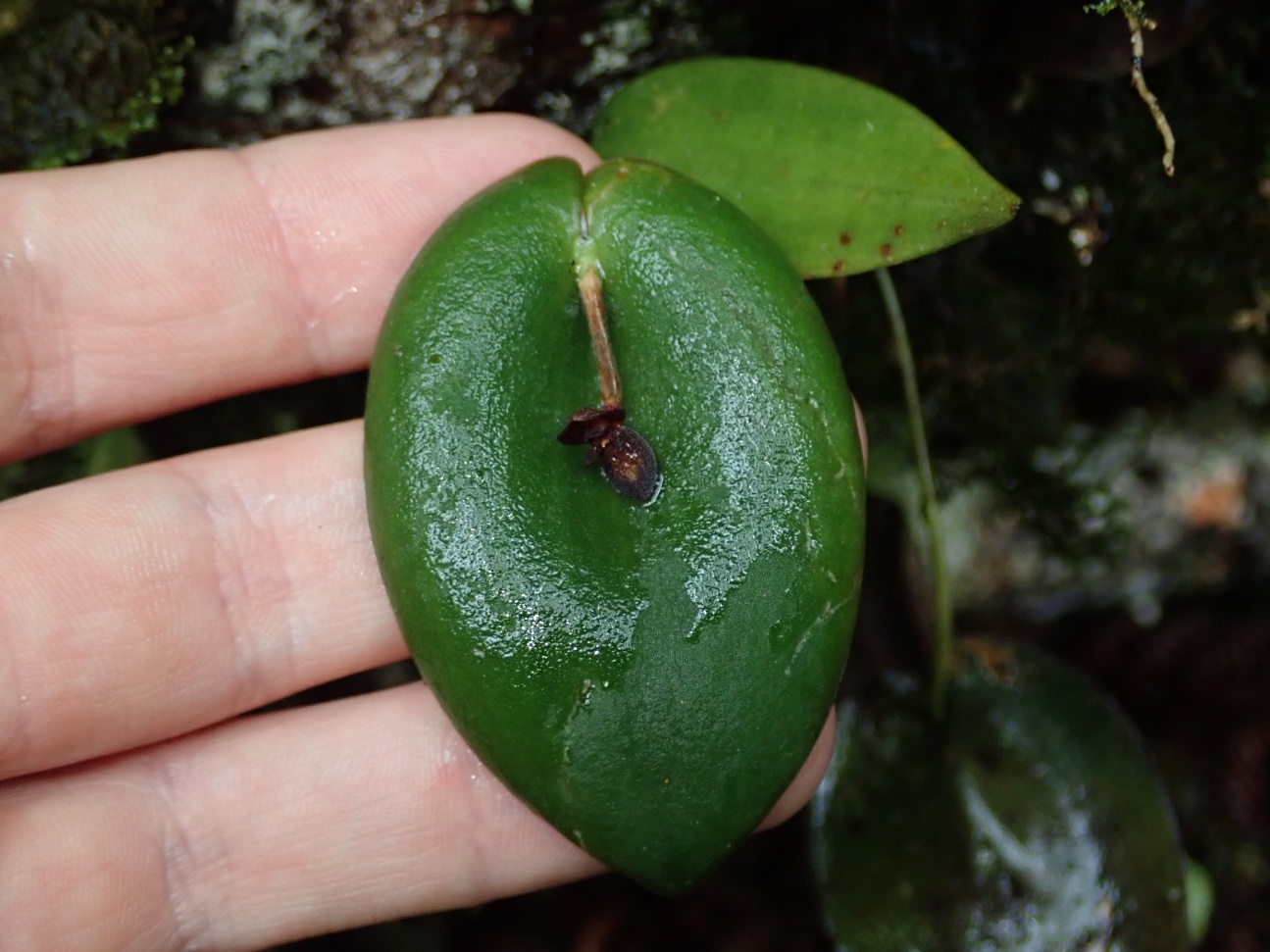
Name of record: *Pleurothallis phymatodea*. Record by: Nolan Exe. iNaturalist user: fern_friend. Date observed: JAN 2022. Date published: MAR 2022. General locality: Ecuador (Diffuse). Latitude: -1.4778427847. Longitude: -78.2381664336. Accuracy: 31.45 km. Geoprivacy: Diffuse. Licensed under CC BY‑NC 4.0.
URL: <https://www.inaturalist.org/observations/108211686>.


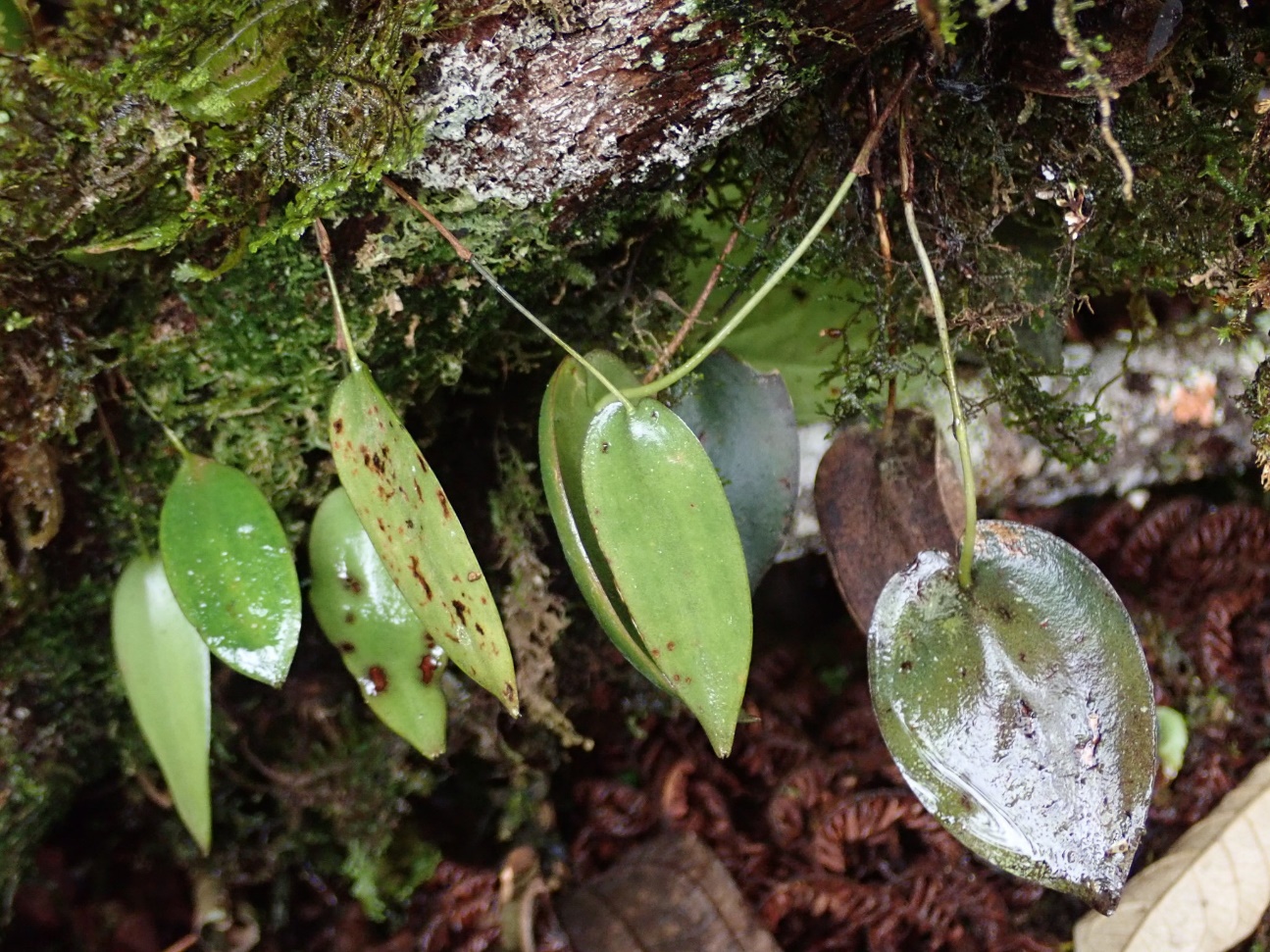


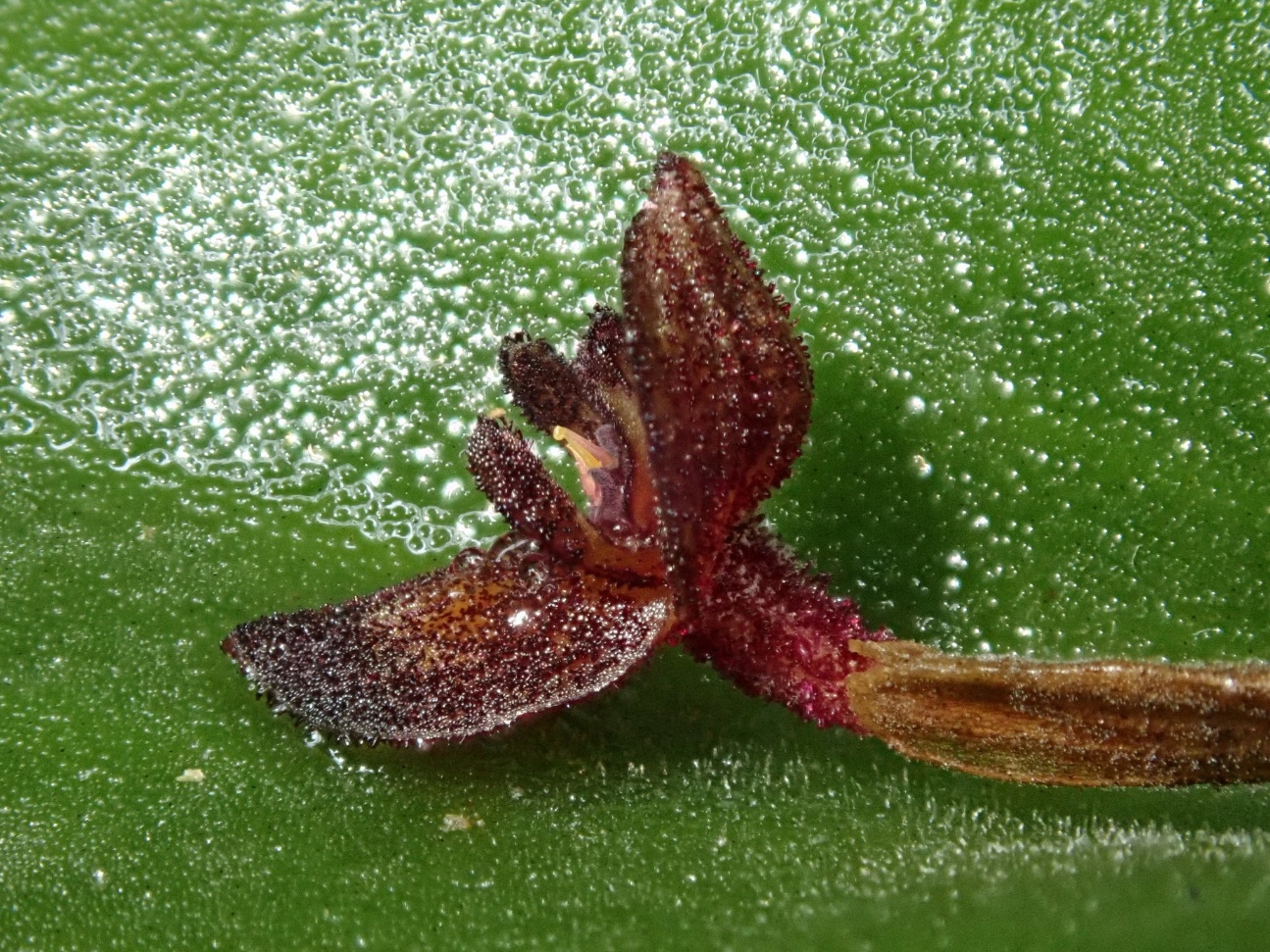

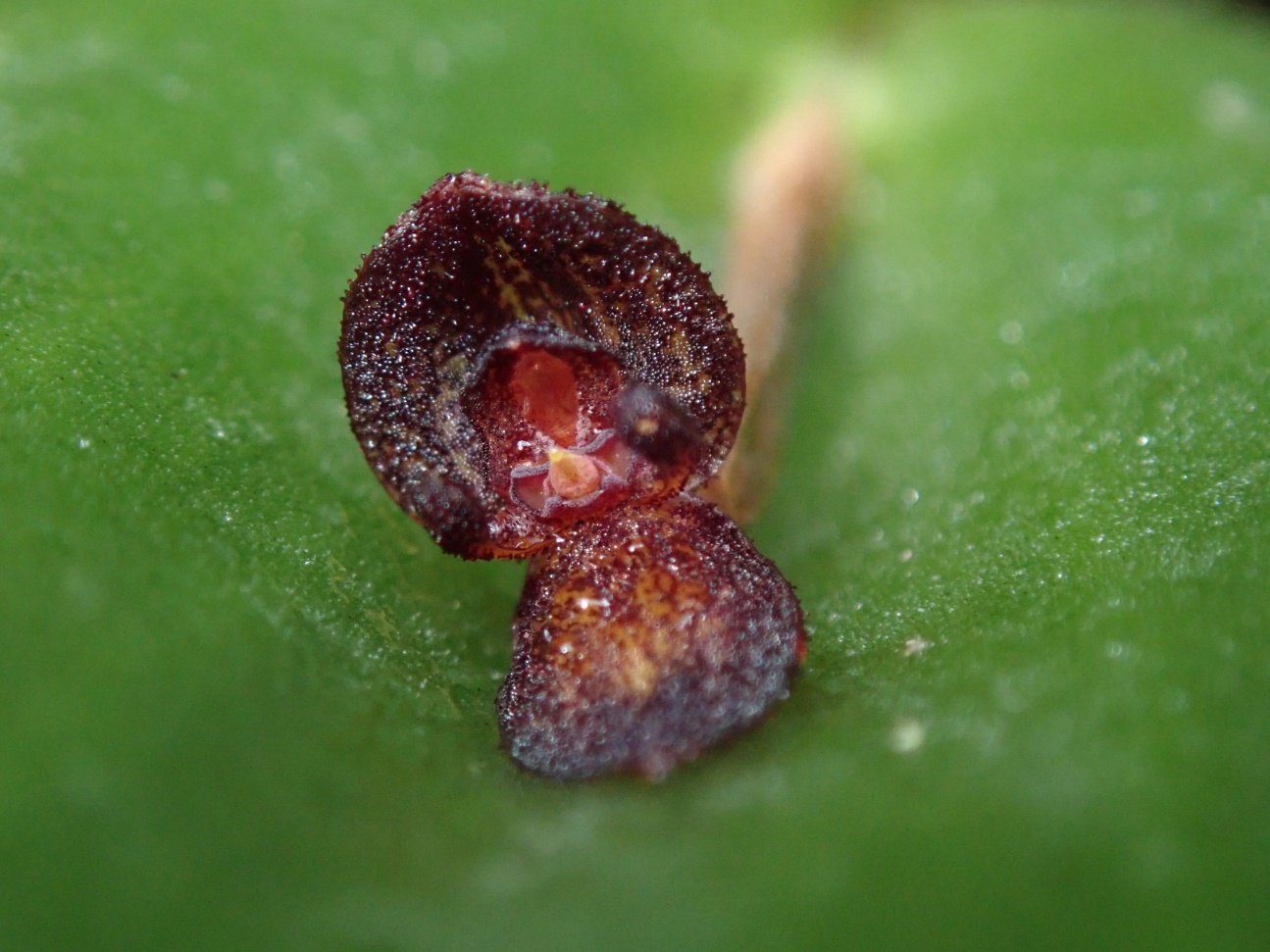

Supplement: Supplementary material 3 — iNaturalist record of Pleurothallis phymatodea [file phytokeys-270-325_article-175070__-s003.docx]
